# Impact of a whole food, plant-based diet on LDL-cholesterol and cardiovascular risk factors in adults with heterozygous familial hypercholesterolemia: a randomized, two-period, two-treatment, crossover, fully controlled feeding trial

**DOI:** 10.1038/s41467-026-73468-4

**Published:** 2026-05-20

**Authors:** Jacob Lessard-Lord, Valérie Guay, Maryka Rancourt-Bouchard, Patrick Couture, Anne Gangloff, Jonatan Blais, André J. Tremblay, Karine Greffard, Jean-François Bilodeau, Iwona Rudkowska, Jean-Philippe Drouin-Chartier

**Affiliations:** 1 Centre Nutrition Santé et Société (NUTRISS), Institut sur la Nutrition et les Aliments Fonctionnels (INAF), Université Laval, Québec, QC Canada; 2 Faculté de Pharmacie, Université Laval, Québec, QC Canada; 3 Centre de Recherche du CHU de Québec-Université Laval, Québec, QC Canada; 4 Département de médecine, Faculté de médecine, Université Laval, Québec, QC Canada; 5 Département de kinésiologie, Faculté de médecine, Université Laval, Québec, QC Canada

**Keywords:** Dyslipidaemias, Risk factors, Metabolic disorders

## Abstract

Heterozygous familial hypercholesterolemia (HeFH) is a genetic disorder that accelerates atherosclerosis and leads to premature cardiovascular diseases (CVD). Whole food, plant-based diets (WFPB) are recommended worldwide for their cardioprotective properties but evidence regarding their effects in HeFH management remains unavailable. This study aims to evaluate the impact of a WFPB, in place of a standard American diet (SAD), on LDL-cholesterol (LDL-C) (primary outcome) and other CVD risk factors among adults with HeFH. In this randomized, two-period, two-treatment, crossover, controlled feeding trial, 50 adults with genetically confirmed HeFH, free of cholesterol-lowering medication, consumed a WFPB and a SAD for 4 weeks each in a random order, under fully controlled, isocaloric feeding conditions. The diets were separated by a two- to four-week washout period. LDL-C and other CVD risk factors were measured at the end of each diet. The WFPB induced a clinically significant reduction in LDL-C relative to the SAD ( − 17.9%, 95% CI: −21.7%, −14.3%; *P* < 0.0001). The study demonstrates the clinical significance of diet therapy in HeFH and supports its re-establishment as a cornerstone in clinical guidelines (clinicaltrials.gov registration: NCT05181553; funding: Canadian Institutes of Health Research).

## Abstract (web summary)

Whole food, plant-based diets are recommended worldwide for their cardioprotective properties but evidence regarding their effects in heterozygous familial hypercholesterolemia remains unavailable. Here the authors show that such a diet induces clinically meaningful benefits in this condition.

## Introduction

Heterozygous familial hypercholesterolemia (HeFH) is an autosomal dominant genetic disorder that disrupts the normal clearance of low-density lipoprotein cholesterol (LDL-C) from the plasma, causing a marked hypercholesterolemia and accelerated atherosclerosis across lifespan^1^. This disease affects approximately 34 million individuals worldwide^2,3^, making it one of the most prevalent genetic disorder causing premature CVDs and deaths^1^. Nine different genes are causative for HeFH, but mutations in the LDL receptor (LDLR) are the most common, accounting for 80–85% of cases^1^. The LDLR genotype is closely associated with inter-individual variability in the phenotypic presentation of the disease, influencing both LDL-C levels and accompanying systemic alterations^4,5^. The residual ability of LDLRs to bind LDL particles is lower in receptor-negative (RN) mutations compared to receptor-defective mutations (RD), which leads to a more severe phenotype and higher CVD risk^6^. HeFH is recognized by the WHO and all major cardiovascular scientific societies as a major public health concern^7^.

Dietary guidelines worldwide emphasize diets low in red and processed meats, and high in whole plant foods, as such patterns broadly improve systemic metabolism and support cardiovascular health^8^. Numerous prospective cohort studies have shown that such dietary patterns are associated with a lower risk of CVD in both the general population and in individuals with a high genetic susceptibility to CVD^9–12^. In the shorter term, randomized controlled trials (RCTs) have shown that such dietary patterns positively affect CVD risk factors, including atherogenic plasma lipids^13–15^. Notably, a meta-analysis of RCT of red meat consumption in comparison with various comparison diets reported that substituting red meat with high-quality plant protein sources (e.g., legumes, soy, and nuts) leads to more favorable changes in blood lipids and lipoproteins^16^.

However, dietary intervention studies conducted to date among individuals with HeFH have focused on the effects of dietary supplements or macronutrient modifications on LDL-C, offering limited insight into how adherence to a healthy dietary pattern—as recommended in contemporary guidelines—may benefit cardiovascular risk in this population^17,18^. In HeFH management, the lack of evidence-based dietary guidelines poses a challenge in its own right, with repercussions on other aspects of care. A systematic review of qualitative studies on treatment adherence in HeFH highlighted that the effectiveness of cholesterol-lowering medications has contributed to a diminished perception of the importance of adopting a healthy diet, making the drug-centered approach a recognized barrier to healthy eating^19^. Individuals with HeFH have reported engaging in unfavorable dietary behaviors due to this misconception^19^, which may exacerbate cardiovascular risk through mechanisms not targeted by cholesterol-lowering medications. Generating high-quality data on the cardioprotective effects of diet in HeFH could help correct this misconception and re-establish the central role of diet in CVD prevention for this population.

This randomized, two-period, two-treatment, crossover, fully controlled feeding, efficacy trial aimed to evaluate the impact of a whole food plant-based diet (WFPB), compared with a standard American diet (SAD), on plasma LDL-C levels (primary outcome), other cardiovascular risk factors and estimated 10-year CVD risk in adults with genetically confirmed HeFH. We hypothesized that, compared to the SAD, the WFPB would reduce LDL-C levels and improve additional cardiovascular risk factors, thereby lowering the estimated 10-year CVD risk. We also examined whether unmodifiable factors—such as LDLR genotype, sex and age—influenced diet-induced changes in LDL-C, assessed the cholesterol-lowering mechanisms of diet, and investigated metabolic correlates of the dietary response. Finally, to support the translation of our findings into clinical practice, we compared the impact of the diets on perceived appetite sensations and their general appreciation by the participants^20^.

## Results

At the time of reporting this paper, the trial had been completed.

### Participant characteristics at baseline

**Fig. 1:**
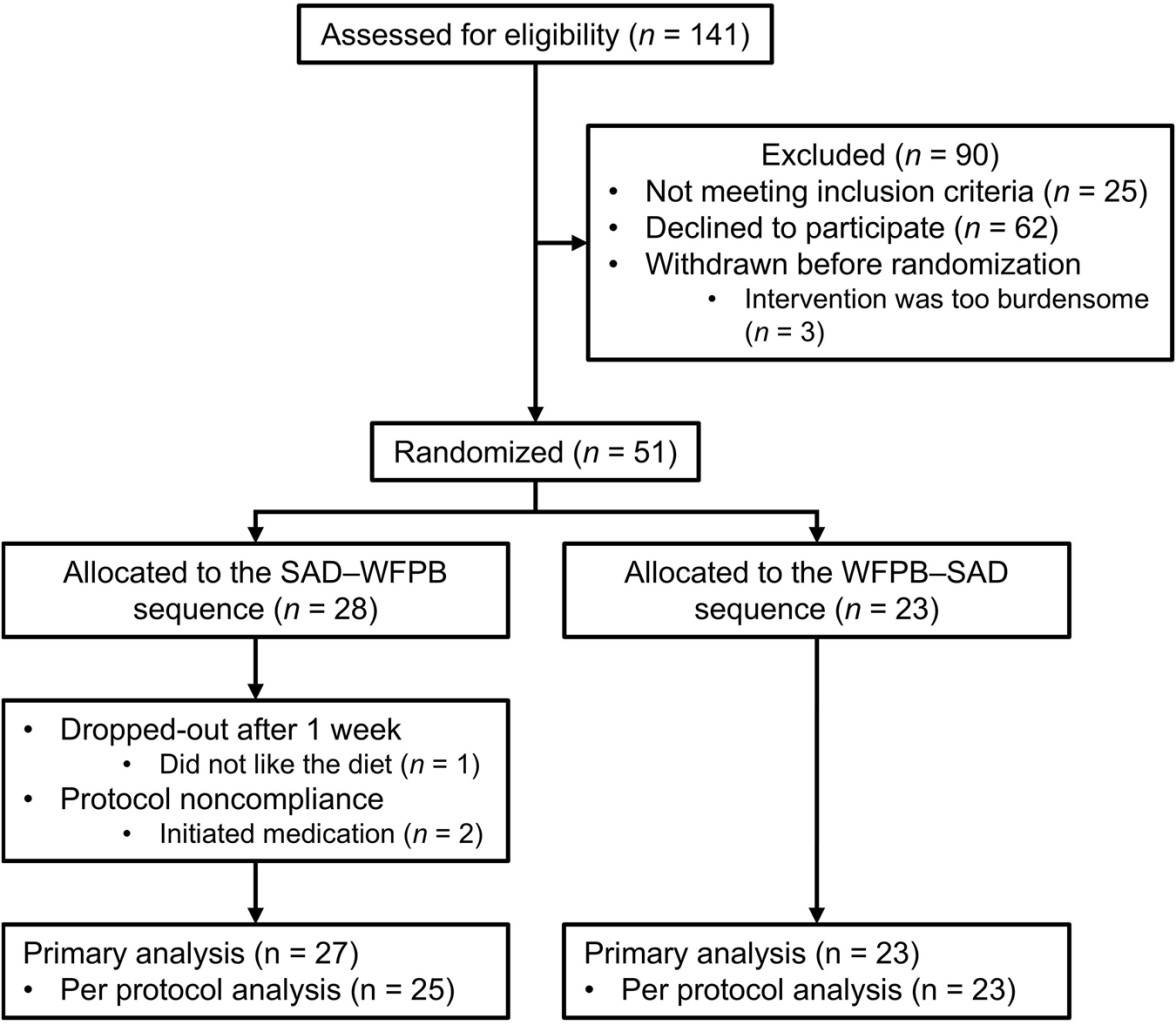
CONSORT (Consolidated Standards of Reporting Trials) flow diagram. SAD: Standard American diet, WPFB: Whole food, plant-based diet.

A total of 51 adults with HeFH were randomized into the SAD-WFPB (*n* = 28) or the WFPB-SAD (*n* = 23) diet sequences (Fig. 1). One participant dropped out during the first week of the trial and could therefore not be included in the analytical models. Additionally, two participants began medications with potential effects on lipid metabolism (isotretinoin and methylphenidate)^21,22^ during the study. These two individuals were excluded from the dataset used in the per-protocol analyzes. Baseline characteristics of the subjects who completed the study are presented in Table 1. There was no apparent difference between subjects according to diet sequences. Age ranged from 19 to 58 years old, and a similar proportion of females (52%) and males (48%) were included in the RCT. Most of the participants had a RN genotype. A greater proportion of RD carrier were assigned to the SAD-WFPB sequence (37%) compared to the WFPB-SAD sequence (17%). Prior to medication discontinuation for the trial, 22% of participants were taking statin monotherapy, 46% were taking statin and ezetimibe, 24% were using a PCSK9 inhibitor with or without concomitant oral therapy, and 8% were not using cholesterol-lowering medication. Subjects were slightly overweight (BMI mean ± SD: 27.0 ± 5.0). Their lipid profile was characteristic of HeFH and their biochemical parameters and blood pressure were within normal range.

**Table 1:** Characteristics of the 50 participants at baseline according to diet sequence^a^

Characteristics	All (*n* = 50)	SAD–WFPB^e^ (*n* = 27)	WFPB–SAD (*n* = 23)
Age, y	37 ± 12	34 ± 13	39 ± 10
Sex			
Female	26 (52%)	14 (52%)	12 (52%)
Male	24 (48%)	13 (48%)	11 (48%)
LDLR genotype			
Receptor-defective	14 (28%)	10 (37%)	4 (17%)
Receptor-negative	32 (64%)	16 (59%)	16 (70%)
Other^b^	4 (8%)	1 (4%)	3 (13%)
Body mass index, kg/m^2^			
Female	27.5 ± 6.1	28.6 ± 7.0	26.2 ± 4.8
Male	26.4 ± 3.6	26.2 ± 3.8	26.6 ± 3.7
Both sexes	27.0 ± 5.0	27.4 ± 5.7	26.4 ± 4.2
Waist circumference, cm			
Female	88.0 ± 14.6	89.4 ± 17.4	86.4 ± 11.1
Male	91.6 ± 9.2	90.2 ± 9.2	93.2 ± 9.5
Both sexes	89.7 ± 12.3	89.8 ± 13.8	89.7 ± 10.7
Pre-trial cholesterol-lowering medication			
None	4 (8%)	2 (7%)	2 (9%)
Statin monotherapy	11 (22%)	5 (18%)	6 (26%)
Statin and ezetimibe	23 (46%)	12 (44%)	11 (48%)
PCSK9 inhibitor with/without oral therapy	12 (24%)	8 (30%)	4 (17%)
Lipid profile			
Total cholesterol, mmol/L	8.13 ± 1.91	8.02 ± 2.00	8.26 ± 1.82
LDL-C, mmol/L^c^	6.17 ± 1.77	6.12 ± 1.87	6.23 ± 1.70
LDL-C_Lp(a) corr._, mmol/L	6.03 ± 1.78	5.97 ± 1.84	6.10 ± 1.75
HDL-C, mmol/L	1.36 ± 0.30	1.34 ± 0.28	1.38 ± 0.34
Triglycerides, mmol/L	1.45 ± 0.96	1.48 ± 1.01	1.42 ± 0.94
ApoA1, g/L	1.45 ± 0.21	1.45 ± 0.22	1.45 ± 0.21
ApoB, g/L	1.68 ± 0.40	1.64 ± 0.41	1.72 ± 0.40
Lp(a), nmol/L	69.3 ± 84.0	72.6 ± 94.1	65.4 ± 72.2
Biochemical parameters			
C-reactive protein, mg/L^d^	1.67 ± 1.86	1.59 ± 1.71	1.77 ± 2.07
Fasting glucose, mmol/L	5.16 ± 0.50	5.15 ± 0.51	5.16 ± 0.51
Fasting insulin, pmol/L	57.1 ± 26.8	55.8 ± 25.1	58.7 ± 29.0
HOMA-IR	2.23 ± 1.16	2.17 ± 1.04	2.29 ± 1.30
HbA1c, %	5.21 ± 0.30	5.18 ± 0.30	5.23 ± 0.30
Blood pressure, mm Hg			
Systolic	112 ± 10	113 ± 7	111 ± 13
Diastolic	69 ± 9	70 ± 8	68 ± 9
Ten-year risk of CVD event according to FH risk score (%)	6.77 ± 6.93	6.03 ± 6.83	7.62 ± 7.11

^a^Data are presented as mean ± standard deviation or count (percent).

^b^Unknown LDLR mutation or non-LDLR mutation (e.g., APOB mutation).

^c^LDL-C was not measured for a single subject.

^d^A subject with an extreme value (41.8 mg/L) was excluded for the descriptive statistics.

^e^SAD: Standard American diet, WFPB: Whole food, plant-based diet.

### Protocol adequacy

Validation metrics of protocol adequacy are presented in Supplementary Table 1. Subjects reported a very high compliance to both experimental diets (>99%), without evidence of a difference in compliance between diets. Energy intake, post-intervention body weight and visceral adipose tissue mass were similar after the two dietary interventions. However, the participants lost a statistically significant amount of weight during both 4-week dietary interventions (mean ± SEM, –0.82 ± 0.22% for SAD and –1.30 ± 0.22% for WFPB). The weight reduction during the WFPB was statistically stronger than during the SAD (∆_absolute_ = –0.48%, 95% CI = –0.92% to –0.05%; *P* = 0.03). Mean ± SD between-diet washout duration was 27 ± 19 days.

### Primary outcome

In intention-to-treat analyzes (Table 2), LDL-C (primary outcome) was decreased by 1.28 mmol/L (95% CI = –1.55 to –1.02; *P* < 0.0001) after 4 weeks of WFPB compared to SAD, which corresponds to a relative reduction of 17.9% (95% CI = –21.7% to –14.3%; *P* < 0.0001). There was substantial inter-individual variability in LDL-C responses to WFPB (Supplementary Fig. 1). Five participants (10% of the sample) experienced LDL-C reductions of ≥30%, while an equal number showed increases in LDL-C on the WFPB compared to the SAD. Among those with increased LDL-C levels, one participant initiated isotretinoin treatment during the study. Notably, only two of these 5 individuals also showed a concurrent increase in ApoB levels (Supplementary Fig. 1). In per-protocol analyzes (Supplementary Table 2), similar results in terms of mean range of response were observed. LDL-C levels were 1.34 mmol/L lower after WFPB than after SAD (95% CI = –1.59 to –1.08; *P* < 0.0001), while the relative difference was –18.6% (95% CI = –22.1% to –15.0%; *P* < 0.0001).

**Table 2:** Outcome levels at the end of the 4-week dietary interventions, analyzed with intention to treat (*n* = 50)

Outcomes	Mean ± SEM		WFPB–SAD difference, mean (95% CI)		*P* value
	SAD	WFPB	Absolute	Relative	
LDL-C, mmol/L (primary outcome)	7.14 ± 0.29	5.86 ± 0.29	–1.28 (–1.55, –1.02)	–17.9% (–21.7%, –14.3%)	<0.0001
Total cholesterol, mmol/L	9.13 ± 0.3	7.65 ± 0.3	–1.48 (–1.74, –1.21)	–16.2% (–19.1%, –13.3%)	<0.0001
LDL-C_Lp(a) corr_., mmol/L	6.99 ± 0.29	5.7 ± 0.29	–1.29 (–1.55, –1.03)	–18.5% (–22.2%, –14.7%)	<0.0001
HDL-C, mmol/L^a^	1.29 ± 0.04	1.18 ± 0.04	–0.11 (–0.16, –0.06)	–8.5% (–12.4%, –4.7%)	<0.0001
Triglycerides, mmol/L^a,b^	1.53 ± 0.13	1.32 ± 0.1	–	–9.5% (–15.3%, –3.4%)	0.004
Non-HDL-C, mmol/L	7.84 ± 0.3	6.47 ± 0.3	–1.37 (–1.62, –1.12)	–17.5% (–20.7%, –14.3%)	<0.0001
ApoA1, g/L	1.36 ± 0.03	1.25 ± 0.03	–0.10 (–0.14, –0.07)	–7.4% (–10.3%, –5.1%)	<0.0001
ApoB, g/L	1.89 ± 0.06	1.61 ± 0.06	–0.28 (–0.34, –0.22)	–14.8% (–18.0%, –11.6%)	<0.0001
Lp(a), nmol/L^a^	69.1 ± 18.5	70.7 ± 18.5	1.6 (–2.4, 5.6)	2.3% (–3.5%, 8.1%)	0.42
C-reactive protein, mg/L^b^	1.62 ± 0.26	1.68 ± 0.28	–	–2.4% (–20.2%, 19.5%)	0.81
Fasting glucose, mmol/L	5.02 ± 0.07	5.04 ± 0.07	0.02 (–0.07, 0.12)	0.4% (–1.4%, 2.4%)	0.63
Fasting insulin, pmol/L	53.0 ± 2.8	54.3 ± 2.8	1.4 (–2.8, 5.6)	2.6% (–5.3%, 10.6%)	0.52
HbA1c, %	5.19 ± 0.04	5.16 ± 0.04	–0.03 (–0.07, 0.01)	–0.6% (–1.3%, 0.2%)	0.14
Blood pressure, mm Hg					
Systolic	112.5 ± 1.3	110.7 ± 1.3	–1.7 (–3.2, –0.3)	–1.5% (–2.8%, –0.3%)	0.02
Diastolic^a^	69.4 ± 1.2	67 ± 1.2	–2.4 (–4.1, –0.7)	–3.5% (–5.9%, –1.0%)	0.008
Ten-year risk of CVD event, %^b^	9.00 ± 1.40	7.38 ± 1.04	–	–11.9% (–19.4%, –3.6%)	0.007

Statistical significance was assessed with linear mixed models adjusted for BMI at the end of each diet (kg/m^2^), weight change during each intervention (%), and diet sequence (SAD-WFPB, WFPB-SAD). Ten-year risk CVD model was not adjusted for age and sex, since these parameters were used to calculate this outcome. *P* values were obtained using two-sided test and were not adjusted for multiple comparison.

^a^A subject had a high influence on the regression which led to a violation of the linear regression postulates (different subject for each outcome). Statistical analysis was re-done without this subject and presented in Table S5. Nevertheless, this violation of the regression postulates did not change the statistical significance of the results.

^b^Analyses were performed on log-transformed data. In such cases, unadjusted mean ± SEM are presented and differences between diets are only presented as percentages and were estimated as 100 × exponential (mean difference of log values) − 100.

### Exploratory outcomes

#### Pre-specified exploratory analyses

Total cholesterol, non-HDL-C, and ApoB all decreased significantly by over 14%, while triglycerides declined by nearly 10%, and HDL-C and ApoA1 by approximately 7% (Table 2). There was no evidence of an effect of diet on Lp(a) levels. Accordingly, results observed for corrected LDL-C_Lp(a) corr._ were similar to those of LDL-C. Compared to the SAD, the WFPB did not impact CRP levels, nor any biomarkers of glucose homeostasis (fasting glucose, fasting insulin and HbA1c). Nevertheless, both systolic and diastolic blood pressure were decreased by approximately 2 mm Hg by the WFPB compared to SAD. Overall, the estimated 10-year risk of CVD event was lower after WFPB than after SAD. Hence, relative to SAD, WFPB decreased the estimated 10-year risk of CVD by 11.9% (95% CI = –19.4% to –3.6%; *P* = 0.007).

In per-protocol analyzes (Supplementary Table 2), similar results in terms of mean range of response were observed for most outcomes. The main models for HDL-C, triglycerides, Lp(a), and diastolic blood pressure violated linear regression assumptions due to a single influential participant. These analyzes were repeated excluding this participant and yielded results similar to the main analyzes in both the intention-to-treat (Supplementary Table 3) and per-protocol (Supplementary Table 4) approaches.

We found no evidence that sex, LDLR genotype, or age (non-pre-specified) modified the effect of the WFPB compared to the SAD on either LDL-C or ApoB levels (Table 3 (intention to treat) and Supplementary Table 5 (per protocol)). There was no indication that diet sequence influenced the effect of WFPB compared to SAD on the outcomes (Supplementary Table 6).

**Table 3:** Effect of dietary intervention on LDL-C and ApoB after stratification by unmodifiable factors, analyzed with intention to treat (*n* = 50)

Stratification	Stratification level	*n*	LDL-C				ApoB			
			WFPB–SAD relative difference, mean (95% CI)	*P* value	*P* value for interaction		WFPB–SAD relative difference, mean (95% CI)	*P* value	*P* value for interaction	
					Stratified	Continuous			Stratified	Continuous
Sex^a^										
	Female	26	–16.7% (–21.2%, –12.1%)	<0.0001	0.72	---	–15.1% (–18.9%, –11.2%)	<0.0001	0.46	---
	Male	24	–19.7% (–25.3%, –14.0%)	<0.0001			–14.0% (–18.7%, –9.2%)	<0.0001		
LDLR genotype^a^										
	Receptor–defective	14	–18.6% (–24.7%, –12.4%)	<0.0001	0.39	---	–14.4% (–19.7%, –9.0%)	<0.0001	0.78	---
	Receptor–negative	32	–17.0% (–21.7%, –12.3%)	<0.0001			–14.5% (–18.5%, –10.5%)	<0.0001		
Age										
	<40 years old	27	–18.0% (–23.3%, –12.7%)	<0.0001	0.55	0.14	–14.6% (–19.1%, –10.2%)	<0.0001	0.58	0.22
	≥ 40 years old	23	–17.9% (–22.6%, –13.1%)	<0.0001			–14.6% (–18.6%, –10.6%)	<0.0001		

Statistical significance was assessed with linear mixed models adjusted for BMI at the end of each diet (kg/m^2^), weight change during each intervention (%), and diet sequence (SAD-WFPB, WFPB-SAD). Models also included the stratification variable and the interaction term between LDL-C/ApoB and the stratification variable. *P* values were obtained using two-sided test and were not adjusted for multiple comparison.

^a^These stratified analyses were pre-specified for LDL-C.

Marked differences in perceived appetite sensations were observed between the diets at the end of the interventions (Table 4). Before meal consumption, both female and male participants felt fuller after WFPB than SAD, while only male participants reported a statistically significant decrease in desire to eat and hunger, which translated into a significantly lower overall appetite score. However, no significant between-sex interaction was observed. After meal consumption, for both female and male participants, fullness was increased and desire to eat, prospective food consumption and appetite score were decreased with WFPB compared to SAD. Relative to SAD, WFPB induced a decrease in hunger sensation only in male participants. Significant statistical between-sex interactions were observed for desire to eat and hunger, with the effect being more pronounced in male than in female participants.

**Table 4:** Appetite sensation difference after the two 4-week dietary interventions assessed with visual analog scales, analyzed per-protocol (*n* = 47)^a^

Appetite sensations	Females (*n* = 25)		Males (*n* = 22)		*P* value for between-sex interaction
	WFPB–SAD relative difference mean (95% CI)	*P* value	WFPB–SAD relative difference mean (95% CI)	*P* value	
Appetite ratings before meals					
Desire to eat	–9.8% (–20.9%, 1.0%)	0.09	–12.8% (–22.8%, –2.7%)	0.01	0.55
Hunger	–7.9% (–19.2%, 3.4%)	0.18	–13.1% (–23.8%, –2.4%)	0.02	0.42
Fullness	20.4% (2.7%, 38.2%)	0.03	26.6% (4.1%, 49.0%)	0.02	0.89
Prospective food consumption	–2.7% (–12.1%, 6.4%)	0.57	–6.9% (–15.5%, 1.7%)	0.12	0.45
Appetite score	–7.4% (–16.3%, 1.4%)	0.11	–10.5% (–18.8%, –2.3%)	0.01	0.49
Appetite ratings after meals					
Desire to eat^b^	–24.6% (–45.1%, 3.3%)	0.09	–54.7% (–67.6%, –35.7%)	<0.0001	0.03
Hunger^b^	–19.6% (–41.5%, 10.4%)	0.19	–49.7% (–64.0%, –28.5%)	0.0001	*0.05*
Fullness	11.7% (4.6%, 18.8%)	0.002	11.4% (3.0%, 19.5%)	0.008	0.84
Prospective food consumption^b^	–31.1% (–48.4%, –8.5%)	0.01	–39.3% (–55.0%, –16.7%)	0.002	0.55
Appetite score^b^	–23.1% (–38.0%, –4.7%)	0.02	–41.7% (–53.5%, –25.8%)	<0.0001	0.09
Satiety quotient	5.1% (–10.8%, 20.9%)	0.53	1.7% (–19.0%, 22.3%)	0.87	0.75
Meal appreciation	–6.5% (–16.2%, 3.1%)	0.20	–1.3% (–11.0%, 8.4%)	0.80	0.48

Statistical significance was assessed with linear mixed models adjusted for meal (breakfast, lunch, and dinner), weekday (Monday, Tuesday, Wednesday, Thursday, Friday, Saturday, Sunday), daily energy intake (kcal), BMI (kg/m^2^), and diet sequence (SAD-WFPB, WFPB-SAD).

^a^A subject did not complete the visual analog scales. *P* values were obtained using two-sided test and were not adjusted for multiple comparison.

^b^Analyses were performed on log-transformed data. In such cases, differences between diets are presented as percentages and were estimated as 100 × exponential (mean difference of log values) − 100.

In addition, changes between the first and last week of each dietary intervention in perceived appetite sensations were assessed (Supplementary Table 7). No differences were observed for appetite ratings before meals, regardless of sex. However, after meals, appetite sensations were modulated between the first and last week of the intervention. First, only during the SAD intervention, desire to eat, hunger and overall appetite score after meals increased over time for male participants, while it decreased for female participants (Supplementary Fig. 2).

At the end of each diet, no difference was observed between diets regarding meal appreciation (Supplementary Fig. 3). However, the appreciation of the SAD meals tended to decline over time for both female and male participants. In contrast, for WFPB meals, appreciation increased for males whereas no statistically significant change was observed in females (Supplementary Fig. 2).

#### Post hoc exploratory analyses

There was no evidence of a statistically significant difference at the end of the WFPB and the SAD in the proportion of participants whose LDL-C levels remained above the thresholds warranting FH investigation according to the Simplified Canadian Definition for FH (≥4.50 mmol/L for those aged 18 to 40 years and >5.00 mmol/L for those aged over 40 years), with corresponding proportions of 79.2% and 87.5% (*P* = 0.13).

No evidence of an effect of the WFPB compared with the SAD was observed for plasma PCSK9 levels (Table 5). Among the surrogate markers of cholesterol synthesis, lathosterol- and lanosterol-to-cholesterol ratios were increased by WFPB compared to SAD, while the desmosterol-to-cholesterol ratio was not impacted by the dietary interventions. In contrast, two indirect markers of cholesterol absorption, campesterol- and cholestanol-to-cholesterol ratios, were decreased following WFPB compared to SAD. The β-sitosterol-to-cholesterol ratio was similar after both diets. Overall, the lathosterol-to-cholestanol ratio, which reflects the balance between cholesterol synthesis and absorption, was increased by 15.8% (95% CI = 8.3% to 24.2%; *P* < 0.0001) after WFPB relative to SAD. Regarding appetite hormones, no evidence of statistically significant effects of the WFPB relative to the SAD was observed on fasting total GLP-1 (3.4%, 95% CI = –0.6% to 7.4%; *P* = 0.09) and peptide YY (–2.6%, 95% CI = –7.0% to 1.7%; *P* = 0.23).

**Table 5:** PCSK9, noncholesterol sterols, and appetite hormones levels at the end of the 4-week dietary interventions, analyzed per-protocol (*n* = 48)

Parameters	Mean ± SEM		WFPB–SAD difference, mean (95% CI)		*P* value
	SAD	WFPB	Absolute	Relative	
PCSK9, ng/mL^a^	368 ± 26	358 ± 28	–	–1.6% (–8.7%, 6.0%)	0.67
Noncholesterol sterols ratio to cholesterol (100 µmol/mmol)					
Surrogates of cholesterol synthesis					
Desmosterol	14.5 ± 0.6	14.3 ± 0.6	–0.20 (–1.20, 0.80)	–1.4% (–8.3%, 5.5%)	0.67
Lathosterol	65.4 ± 3.6	70.6 ± 3.6	5.19 (0.31, 10.07)	7.9% (0.5%, 15.4%)	0.04
Lanosterol	22.2 ± 1.3	24.9 ± 1.3	2.63 (–0.09, 5.36)	11.8% (–0.4%, 24.1%)	0.06
Surrogates of cholesterol absorption					
Campesterol	99.9 ± 4.9	93.6 ± 4.9	–6.29 (–12.22, –0.36)	–6.3% (–12.2%, –0.4%)	0.04
β-Sitosterol^a^	68.1 ± 3.2	69.1 ± 3.3	–	2.9% (–2.0%, 8.1%)	0.24
Cholestanol	59.7 ± 2.0	54.7 ± 2.0	–5.10 (–8.20, –2.00)	–8.5% (–13.7%, –3.4%)	0.002
Lathosterol to cholestanol ratio (µmol/µmol)	1.20 ± 0.09	1.39 ± 0.09	0.19 (0.10, 0.29)	15.8% (8.3%, 24.2%)	<0.0001
Appetite hormones					
Total GLP-1, pmol/L	20.9 ± 2.9	21.6 ± 2.9	0.71 (–0.12, 1.54)	3.4% (–0.6%, 7.4%)	0.09
Peptide YY, pg/mL	28.8 ± 0.7	28.1 ± 0.7	–0.76 (–2.02, 0.49)	–2.6% (–7.0%, 1.7%)	0.23

Statistical significance was assessed with linear mixed models adjusted for BMI at the end of each diet (kg/m^2^), weight change during each intervention (%), and diet sequence (SAD-WFPB, WFPB-SAD). *P* values were obtained using two-sided test and were not adjusted for multiple comparison.

^a^Analyses were performed on log-transformed data. In such cases, unadjusted mean ± SEM are presented and differences between diets are only presented as percentages and were estimated as 100 × exponential (mean difference of log values) − 100.

No evidence of association between any potential correlates and LDL-C response to the WFPB was found (Supplementary Table 8). For ApoB response, each 1 pmol/L-increment in fasting total GLP-1 was associated with a –0.15% decrease in ApoB diet-induced changes (95% CI = –0.31% to –0.01%, *P* value not adjusted for multiple comparison = 0.04). The partial *R*^2^ associated with fasting total GLP-1 was 12.6%.

## Discussion

This randomized, crossover, two-period, two-treatment, fully controlled feeding efficacy trial evaluated the 4-week impact of a WFPB, compared with a SAD, on plasma LDL-C levels, other cardiovascular risk factors, and the estimated 10-year risk of CVD in adults with genetically confirmed HeFH. The WFPB was primarily based on key recommendations for healthy food choices outlined in the 2019 Canada’s Food Guide, but also aligned with core principles of the 2020 Dietary Guidelines for Americans, the Mediterranean diet, and the Portfolio diet. Similarly, the SAD was designed to reflect the current dietary pattern of adults in the Province of Québec (Canada), but shared many characteristics with the average North American diet. Compared to the SAD, the WFPB significantly decreased LDL-C (primary outcome) by 17.9%. Additionally, the WFPB led to improvements in other atherogenic lipid fractions and blood pressure. Overall, transitioning from the SAD to the WFPB resulted in an 11.9% relative reduction in the estimated 10-year risk of CVD event, as assessed by the FH Risk Score. In addition, the WFPB diet reduced intestinal cholesterol absorption and improved perceived appetite sensations compared to the SAD. Overall, this study demonstrates that transitioning from a SAD to a WFPB diet has a clinically meaningful effect in adults with HeFH in the context of CVD prevention.

This study is the first to test the effect of a dietary pattern low in red and processed meat and high in whole plant-based foods on CVD risk factors in adults with genetically confirmed HeFH. The 18% decrease in LDL-C and 15% reduction in ApoB observed closely mirror the findings of a meta-analysis of dietary intervention studies on the Portfolio diet–specifically designed to lower LDL-C^23^–which reported reductions of 17% and 15% in LDL-C and ApoB across seven RCTs in individuals with hyperlipidemia, including phenotypic, but not genetically confirmed HeFH^24^. As such, our study suggests that dietary modification can be as effective at reducing LDL-C and ApoB in individuals with genetically confirmed HeFH as it is in those without the condition. However, we acknowledge that dietary therapy alone is insufficient to achieve contemporary target levels of LDL-C in individuals with HeFH (i.e., <2.50 mmol/L)^17^.

In addition to reductions in atherogenic plasma lipids, the WFPB also decreased HDL-C and its associated ApoA1 by –8.5% and –7.4%, respectively, compared to the SAD. Similar reductions have been reported in previous studies testing the effects of plant-based diets on plasma lipids^13^. One possible explanation for this phenomenon is the lower intake of saturated fatty acids during the WFPB (6.5% of daily energy) compared to the SAD (12.0% of daily energy), as saturated fatty acids have been shown to increase HDL-C as well as LDL-C^25,26^. Importantly, the total cholesterol-to-HDL-C ratio was lower with the WFPB than with the SAD, indicating that atherogenic lipids—causally linked to CVD risk^27^—were reduced to a greater extent than HDL-C, whose causal link with CVD remains debatted^28^. Future studies could test WFPB dietary patterns with alternative fat profiles, such as greater contributions from non-animal sources of saturated fat (e.g., cocoa and coconut fat), to determine whether LDL-C and ApoB benefits can be maintained while attenuating decreases in HDL-C and ApoA1. Likewise, future studies could examine whether a WFPB diet with a different macronutrient distribution, such as a higher content of plant-derived unsaturated fatty acids similar to that tested in the PREDIMED trial^29^, would confer greater benefits on plasma lipids and other CVD risk factors than those observed in the present study.

Overall, the substantial reductions in atherogenic lipids and lipoproteins and blood pressure observed in our study led to a reduction in the estimated 10-year CVD risk, calculated using the validated FH Risk Score^30^. Specifically, relative to the SAD, the WFPB reduced by 11.9% the estimated 10-year CVD risk. Given that our trial does not allow inference as to whether this estimated risk reduction would translate into a lower CVD incidence over time, studies evaluating the impact of, or prospective associations with, adherence to a WFPB diet on CVD risk in HeFH are still needed. Nevertheless, the estimated reduction in CVD risk observed here is consistent with that associated with high versus low adherence to the Portfolio diet in a pooled analysis of three large prospective cohort studies, and with that associated with high adherence to healthful plant-based dietary patterns^12,31^.

The decision to test the effect of diet without concomitant use of cholesterol-lowering medication was driven by the objective of rigorously isolating the full potential of diet in improving LDL-C and other CVD risk factors in genetically confirmed HeFH—a question that had not been addressed to date. The absence of cholesterol-lowering medication use among participants may limit the generalizability of our findings, given the central role of these medications in HeFH management. Future studies should determine the incremental LDL-C–lowering effect of diet on top of cholesterol-lowering medication, as well as whether medication type (e.g., statin, ezetimibe, PCSK9 inhibitor), alone or in combination, modifies the effect of diet on LDL-C lowering. Nevertheless, nearly all prior studies using an adequate 2×2 factorial design have reported additive benefits of diet and statins on atherogenic plasma lipids, meaning that concomitant medication use does not attenuate the effects of diet on LDL-C^32–36^.

We found no evidence that sex, age, or LDLR genotype were associated with the LDL-C or ApoB response to the WFPB. Regarding sex, we did not anticipate effect modification. However, reporting sex-specific results is expected to facilitate translation of the importance of dietary modification into clinical practice—particularly given that male individuals generally show lower adherence to healthy diet principles compared to female individuals. The lack of effect modification by LDLR genotype may pave the way for personalized management approaches. A previous retrospective longitudinal study showed that statin-induced LDL-C reductions were greater in individuals with RD genotype than in those with RN genotype^37,38^. Based on this, one could propose that dietary counseling be particularly emphasized for individuals with RN genotype, given their lower response to statin.

Regarding the cholesterol-lowering mechanisms of diet, measurements of noncholesterol sterols suggest that the WFPB, compared to the SAD, reduced plasma LDL-C levels primarily by decreasing cholesterol absorption. As expected, cholesterol synthesis increased to maintain homeostasis; however, the rise in the lathosterol-to-cholestanol ratio indicates that the reduction in absorption outweighed the increase in synthesis. These findings align with the known effects of increased soluble fiber intake, which forms a gel that traps bile acids, reducing their reabsorption and thereby limiting intestinal cholesterol uptake^39^. This depletion of bile acids promotes hepatic conversion of cholesterol into bile acids, further enhancing reductions in circulating LDL-C^40^. The modulation of cholesterol homeostasis induced by diet is also consistent with that induced by ezetimibe, which lowers LDL-C by inhibiting cholesterol absorption through the Niemann-Pick C1-Like 1 protein^41^. Accordingly, the effect of the WFPB diet on the lathosterol-to-cholestanol ratio further supports the need for comprehensive studies examining how diet interacts with different classes and combinations of cholesterol-lowering medications.

In this study, we observed a reduction in perceived appetite sensations with WFPB compared to SAD. The lower energy density of the WFPB may have contributed to this effect, especially considering that the diets were provided in isocaloric, weight-maintaining conditions. However, no evidence of effect on fasting total GLP-1 was found to provide biological support for these results. Future studies should assess how WFPB diets impact total and active GLP-1 in the postprandial state, since fasting measures may miss meal-stimulated differences relevant to perceived appetite sensations.

Finally, data on participants’ appreciation of the experimental diets provide possible insights into how such a dietary transition could be supported in real-life settings. We hypothesize that the similar appreciation of the WFPB and SAD after each 4-week period may be partly explained by the fact that both diets included typically Canadian meals. This suggests that adapting familiar traditional recipes to fit a WFPB pattern may help overcome common barriers related to taste acceptability and perceptions of WFPB eating, without reducing overall appreciation^42–44^.

The study needs to be interpreted in light of both its strengths and limitations. The main strength lies in its design, which isolated the effect of diet on study outcomes through within-subject comparisons. Still, with each intervention lasting 4 weeks, the findings reflect short-term effects of the diets on LDL-C and other cardiovascular risk factors. Future studies should assess the long-term impact of diet in HeFH, particularly in conjunction with cholesterol-lowering medication, on both plasma lipids and CVD outcomes. Additional trials are also warranted to determine whether a WFPB diet has clinically meaningful effects on CVD risk factors in children and adolescents with HeFH, as well as in older adults aged >60 years, which were not included in the present trial. Second, all participants had genetically confirmed HeFH and carried one of the nine French-Canadian FH-causing variants. This enhances the generalizability of findings to individuals with HeFH from the same population—as this group is particularly affected by HeFH due to a founder effect. However, given that over 2000 FH-causing mutations have been identified worldwide^1^, this may limit the generalizability to more genetically diverse populations. Third, most participants had normal CRP and glucose homeostasis, limiting the potential for improvement with WFPB diet. In addition, changes in these parameters are more consistently observed in cohorts with elevated baseline values and/or with longer interventions and/or meaningful weight loss, which may partly explain why we observed no effect. Fourth, the effect of the dietary interventions on cholesterol metabolism was inferred from indirect markers of cholesterol synthesis and absorption. These findings should be validated using direct assessment methods, such as the sterol balance technique or the dual-isotope method^45^. Fifth, we assessed patient-reported outcomes (appetite sensation and diet appreciation) to facilitate clinical translation^20^. Sixth, given the number of exploratory outcomes assessed, some findings may have occurred by chance. Finally, implications associated with the fact that the trial was conducted without cholesterol-lowering medication are a limitation and have been previously discussed.

In conclusion, this randomized, two-treatment, two-period, crossover, fully controlled feeding efficacy trial demonstrated that a WFPB, emphasizing high intakes of whole plant-based foods and low intakes of red and processed meats, refined grains and sugary drinks, in place of a SAD, reduces LDL-C and improves other cardiovascular risk factors in adults with HeFH. These diet-induced changes translated into a reduction in the estimated 10-year CVD risk. Our findings underscore the clinical significance of diet therapy in HeFH and support its re-establishment as a cornerstone in future clinical guidelines. Further studies are needed to assess the interaction of diet modification alongside cholesterol-lowering medication. Such work will be essential to develop personalized treatment strategies to optimize treatment response in HeFH.

## Methods

The CHU de Québec-Université Laval ethical review committee approved the research protocol. Written informed consent was obtained from all participants. This trial was registered at clinicaltrials.gov (NCT05181553) on December 20th, 2021, with the protocol accessible on this repository. No patients or members of the public were involved in setting the research question or the outcome measures, nor were they involved in the design and implementation of the study. The study was conducted at the Institute of Nutrition and Functional Foods (INAF) of Laval University in Quebec City, Canada, between January 2022 and June 2024. The first participant was enrolled on January 24th, 2022, and the last participant was enrolled on January 18th, 2024. The study is reported in accordance with the Consolidated Standards of Reporting Trials (CONSORT) statement^46^. Participants and study coordinators could not be blinded to the interventions due to their nature. However, laboratory analyzes were conducted in a blinded fashion, and investigators remained blinded to the intervention groups during statistical analyzes until the final analyzes of the primary outcome were completed.

### Study participants

Volunteers were recruited through referral from physician collaborators at the Lipid Clinic of the CHU de Québec-Université Laval in Quebec City, Canada, where >1000 individuals with HeFH are followed. To be eligible, participants had to be adults (18 to 60 years old) with genetically confirmed HeFH. Data on the FH-causing variant and LDLR genotype were obtained from medical records. Premenopausal female participants were required to have a regular menstrual cycle for more than 3 months and postmenopausal female participants were required not to use hormone replacement therapy to be eligible. The age threshold was driven by the requirement to discontinue cholesterol-lowering medication. Because older adults have a higher baseline CVD risk, participants aged ≥60 years were excluded as a risk-mitigation measure based on safety considerations related to temporary treatment withdrawal. Other exclusion criteria included conditions that exacerbate CVD risk (e.g., homozygous FH, personal history of CVD, diabetes or use of anti-diabetic medication, severe obesity, unstable body weight over the previous 3 months, uncontrolled hypertension, and genetic hypertriglyceridemia), allergies or aversions to components of the experimental diets, and any condition that could interfere with optimal participation in the intervention.

### Study design

This randomized, two-period, two-treatment, crossover, fully controlled feeding efficacy trial was designed to assess the impact of a WFPB, relative to a SAD, on LDL-C concentrations, other cardiovascular risk factors and estimated 10-year CVD risk in adults with HeFH. A crossover design is an efficient approach for estimating treatment effects because each participant serves as their own control, thereby reducing between-participant variability and the required sample size^47^.

After enrollment, participants entered a 4-week run-in period during which those taking lipid-lowering medications were asked to discontinue them for the duration of the trial. A 4-week washout period of cholesterol-lowering medication is sufficient for LDL-C levels to return to untreated values^48–50^. Other medications (e.g., anti-hypertensive drugs, oral contraceptives) were allowed, but doses had to remain constant during the study. Participants were asked not to initiate any new medications during the study.

After the run-in period, participants attended a baseline visit at the INAF Clinical Research Unit, during which they provided fasting plasma samples (collected in EDTA tubes) and completed a self-administered, validated, web-based food frequency questionnaire^51^. They were subsequently randomized to either the SAD–WFPB or WFPB–SAD diet sequence. Randomization was performed by the research coordinators and was stratified by sex (female, male) and LDLR genotype (RN vs. others), using eight blocks of eight participants and a 1:1 allocation ratio, such that each block included equal numbers of females and males and equal numbers of individuals with the RN genotype and other genotypes. Following randomization, participants entered the fully fed intervention periods. Each diet was consumed for 4 weeks (28 days) and was separated by a 2- to 4-week washout period. At the end of each diet period, fasting plasma samples were collected from an antecubital vein using EDTA tubes. Four-week periods of fully controlled feeding are well established to produce clinically significant changes in plasma LDL-C and other outcomes of interest, as shown in numerous previous studies^14,23,52^. Likewise, a 2- to 4-week washout period has also been shown to be sufficient to minimize potential carryover effects between periods^14,23,52^. During the diet phases, participants received all meals and caloric beverages, structured into three meals and one snack per day, based on a 7-day rotating menu. An experienced food technician at the INAF metabolic kitchen prepared the meals, with each ingredient precisely weighed (±0.1 g). The two diets were isocaloric and designed to maintain participants’ weight. Energy needs were estimated using the Harris–Benedict equation and cross-validated with data from the baseline food frequency questionnaire. Diets were then proportionally tailored to match individual energy requirements. Participants visited the INAF Clinical Research Unit three times weekly (Monday, Wednesday, Friday) to collect their meals and had the option to eat on-site under supervision. Body weight was monitored at each visit, and caloric intake was adjusted if a weight change of ±1 kg was observed. Participants were instructed to consume only the provided foods and to consume all meals as supplied. They were also asked to avoid consuming any other foods or caloric beverages, including alcohol, during the dietary interventions and to avoid adding any calorie-containing seasoning or salt to their meals. They were instructed to only drink, in addition to the beverages provided, water or caffeine-free diet beverages and a maximum of 2 cups per day of tea, black coffee or other caffeine-containing diet beverages. They were instructed on which calorie-free seasonings to use if desired. A checklist was provided to the participants to identify foods that they had or had not consumed, and was used to assess compliance to the dietary interventions. Participants were also instructed to indicate in the checklist if they consumed any non-study foods or beverages. Finally, participants were asked to maintain their usual physical activity throughout the entire study and to avoid the use of vitamin supplements or natural health products during the trial.

### Composition of the experimental diets

The WFPB was primarily tailored to align with the key recommendations of the 2019 Canada’s food guide^53^. Consequently, it also closely adhered to fundamental concepts of other heart-healthy dietary guidelines or patterns, including the 2020 Dietary Guidelines for Americans^54^, the Mediterranean diet^55^, and the Portfolio diet^23^ (Table 6). A key distinction from the Mediterranean diet was that canola oil was used for cooking instead of olive oil. Canola oil is locally produced in Canada and contains a similar proportion of monounsaturated fatty acids as olive oil. Likewise, a key distinction from the Portfolio diet was that no phytosterol-fortified foods were included in the experimental diet. The WFPB emphasized high intakes of minimally processed plant foods (e.g., whole and fresh fruits, vegetables, whole grains, and legumes), and low intakes of red and processed meat (Supplementary Note 1). Daily, the menu integrates ≥1 recipe from Canada’s Food Guide official documentation. Daily portions of protein foods, whole grains, fruits, and vegetables followed the recommended ¼-¼-½ plate proportions outlined in Canada’s food guide. In terms of protein quality, plant-based proteins were prioritized over animal proteins. Red meat was consumed only once per week, and 2 days each week were entirely meatless. Homemade recipes were prioritized over commercially prepared foods. Water was the primary beverage in this dietary intervention; to encourage water consumption, participants were provided with reusable water bottles.

**Table 6:** Nutritional composition of the two experimental diets

Daily intake per 2500 kcal	SAD	WFPB
Carbohydrates (% of energy)	50.0 ± 0.0	50.0 ± 0.0
Sugars (g)	157.6 ± 16.2	137.6 ± 18.6
Added sugars (g)	91.5 ± 17.7	38.4 ± 8.7
Total fiber (g)	22.4 ± 1.6	47.5 ± 8.7
Soluble fiber (g)	6.7 ± 1.4	11.3 ± 1.9
Insoluble fiber (g)	15.7 ± 1.4	36.2 ± 6.9
Proteins (% of energy)	15.0 ± 0.0	15.0 ± 0.0
Animal proteins (% of energy)	9.7 ± 0.6	5.6 ± 2.3
Plant proteins (% of energy)	5.3 ± 0.6	9.4 ± 2.2
Lipids (% of energy)	35.0 ± 0.0	35.0 ± 0.0
Saturated fatty acids (% of energy)	12.0 ± 0.0	6.5 ± 1.8
Monounsaturated fatty acids (% of energy)	13.7 ± 1.3	16.5 ± 0.7
Polyunsaturated fatty acids (% of energy)	6.8 ± 1.2	9.8 ± 2.2
Trans fatty acids (% of energy)	1.0 ± 0.4	0.5 ± 0.2
Omega-3 polyunsaturated fatty acids (g)	3.2 ± 1.3	5.3 ± 1.9
Omega-6 polyunsaturated fatty acids (g)	16.1 ± 3.8	22.9 ± 5.0
Phytosterols (mg)	74 ± 36	116 ± 56
Cholesterol (mg)	366 ± 133	203 ± 152
Sodium (mg)	3515 ± 105	2519 ± 285
Animal foods (% of energy)	45 ± 9	18 ± 6
Plant foods (% of energy)	55 ± 9	82 ± 6
Food quantity (g)		
Animal proteins		
Unprocessed red meat	19 ± 33	9 ± 23
Processed meat	54 ± 27	0 ± 0
Poultry	78 ± 42	28 ± 38
Eggs	31 ± 42	32 ± 51
Fish/seafood	13 ± 35	34 ± 43
Dairy products	280 ± 131	131 ± 106
Total	474 ± 133	234 ± 103
Plant proteins		
Soy-based products	0 ± 0	178 ± 90
Legumes	23 ± 21	99 ± 75
Nuts/seeds	19 ± 13	40 ± 20
Total	42 ± 24	318 ± 94
Grains		
Whole	41 ± 41	229 ± 68
Refined	200 ± 61	40 ± 72
Whole fruits	243 ± 74	565 ± 164
Vegetables	401 ± 119	684 ± 263
Sugary drinks	394 ± 163	141 ± 198
Energy density (kcal/g)	1.30 ± 0.12	1.04 ± 0.16
Energy density without beverage (kcal/g)	1.55 ± 0.14	1.12 ± 0.22
Unprocessed or minimally processed foods (% of energy)^a^	31 ± 7	46 ± 7
Ultra-processed foods (% of energy)^a^	47 ± 8	33 ± 10
Mediterranean diet score, /44 pts^b^	22 ± 1	38 ± 1
Healthy Eating Index-2020, /100 pts^c^	50 ± 8	79 ± 3
Healthy Eating Food Index-2019, /80 pts^d^	39 ± 6	71 ± 4

Data are presented as mean ± standard deviation.

^a^According to the NOVA classification.

^b^Calculated as previously described by Goulet et al.^55^.

^c^The Healthy Eating Index-2020 reflects adherence to the 2020 Dietary Guidelines for Americans.

^d^The Healthy Eating Food Index-2019 reflects adherence to the 2019 Canada’s food guide.

The SAD was designed to reflect the current dietary intakes—regarding foods, nutrients, and diet quality—of adults in the Province of Québec (Canada), based on the most recent dietary surveys^56–58^. The purpose of this control diet was to induce, at the end of the 4-week period, a metabolic state that mimics that of an untreated individual at the moment of HeFH diagnosis. This diet was characterized by low consumption of whole fruits and vegetables, with animal proteins—particularly red and processed meats—being the primary protein sources. Grain consumption was predominantly in the form of refined grains. Additionally, the diet included daily servings of ready-to-heat or ready-to-eat foods, considered as ultra-processed according to the Nova classification^59^, along with sugary beverages. By extent, it also shared many characteristics with typical diets elsewhere in North America (e.g., 12% of energy from saturated fat, ~50% of energy from ultra-processed foods).

The two diets were designed so that most meals were matched in terms of type and content, with differences stemming from the choice of ingredients and preparation methods. The menus for both the WFPB and SAD diets are presented in Supplementary Table 9, along with photos of three representative meals from each diet in Supplementary Image 1. The difference in food quality between the two experimental diets influenced their nutritional composition (Table 6). The WFPB contained less saturated fat, cholesterol and sodium, and more dietary fiber, unsaturated fatty acids, and phytosterols compared to the SAD. Despite these differences, both diets were designed to provide the same proportions of energy: 50% from carbohydrates, 15% from protein, and 35% from fat. The nutritional composition of the experimental diets was assessed using the Nutrition Data System for Research software (University of Minnesota).

### Plasma lipids and biochemical analyses

Lipids, C-reactive protein (CRP), glucose, insulin and glycated hemoglobin (HbA1c) were measured in fasting plasma samples collected after the run-in period (i.e., baseline visit) and at the end of each dietary intervention. Total cholesterol, high-density lipoproteins-cholesterol (HDL-C), triglycerides, and glucose were quantified using a Siemens Dimension Vista (Siemens Healthcare Limited, Germany) with proper reagents. LDL-C was calculated using Friedewald’s formula and non-HDL-C was derived by subtracting HDL-C from total cholesterol. Apolipoprotein B (ApoB), apolipoprotein A1 (ApoA1), and CRP were determined by nephelometry using a Siemens Dimension Vista (Siemens Healthineers, Germany). Lipoprotein(a) (Lp(a)) was quantified using a Roche Cobas modular analyzer (Roche Diagnostics, Indianapolis, USA). Since Lp(a)-cholesterol concentration is included within LDL-C by the Friedewald’s formula^60^, we also calculated corrected LDL-C values for Lp(a) cholesterol (LDL-C_Lp(a) corr._) by subtracting Lp(a)-cholesterol (estimated as 30% of Lp(a) mass) from LDL-C, as previously described^61^. Insulin was quantified using a Siemens Centaur XPT (Siemens Healthineers, Germany). HbA1c was measured using a VARIANT II TURBO Hemoglobin Testing System (Bio-Rad Laboratories Inc., Hercules, USA).

### Anthropometry measurements

At baseline and at the beginning and end of each experimental diet, body weight and waist circumference were measured by the same study coordinators for the entire study following standard procedures^62^. Participants’ height was also measured at baseline to enable body mass index (BMI) calculation. At the end of each dietary intervention, visceral adipose tissue was determined by a dual-energy X-ray absorptiometry scan (Prodigy instrument and CoreScan application, enCORE software version 14.1, GE Healthcare, Chicago, USA)^63^.

### Blood pressure assessment

Participants’ systolic and diastolic blood pressure were recorded using an automatic blood pressure monitor (BP Thru, Omron, Kyoto, Japan) attached to their right arm by a trained research nurse after they had remained quietly seated for 10 min. Three consecutive readings were taken with 3-min intervals between each measurement.

### Ten-year CVD risk estimation

Ten-year risk of CVD event was estimated using the validated FH Risk Score^30^. The risk is calculated based on sex, age, HDL-C, Lp(a), and LDL-C levels, hypertension (yes or no) and active smoking (yes or no).

### Appetite hormones and PCSK9 measurements

Quantification in fasting EDTA plasma samples was carried out by ELISA for total glucagon-like peptide-1 (GLP-1) (kit #43-GPTHU-E01, Alpco, Salem, USA), peptide YY (kit #027336, United States Biological, Salem, USA) and PCSK9 (kit #CY-8079, MBL International, Schaumburg, USA) according to the manufacturers’ procedures.

### Noncholesterol sterols measurements

Noncholesterol sterols, which their ratio to cholesterol is a surrogate marker of cholesterol absorption and synthesis^45,64^, were measured in fasting EDTA plasma samples by GC-MS after derivatization. Briefly, these molecules were extracted by mixing 10 µL of plasma with 10 µL of cholesterol-d_6_ from Avanti Research (0.1 mg/mL) as an internal standard, followed by the addition of 25 µL of 99% anhydrous ethanol and 7.6 µL of 8.9 M potassium hydroxide. The mixture was incubated at 70 °C for a period of 3 h to liberate esterified noncholesterol sterols. The reaction was stopped by adding 13.5 µL of hydrochloric acid (5 N), after allowing the tube to cool to room temperature and briefly spinning it to bring down condensation. To remove non-volatile lipids and proteins, 500 µL of HPLC grade acetone was added to each sample, followed by vortexing and centrifugation at 16,000 × *g* for 5 min. The supernatant was transferred and evaporated under a stream of nitrogen. To further reduce matrix interference, a 200 µL ethanol rinse was performed. After mixing and centrifugation under the same conditions (5 min at 16,000 × *g*), a volume of 175 µL was carefully collected and evaporated to dryness prior to derivatization. The derivatization process was carried out by adding 100 µL of pyridine/N-Methyl-N-(trimethylsilyl)trifluoroacetamide and 1% Trimethylchlorosilane mixture (1:9, v/v) followed by an incubation at 37 °C for 30 min. GC-MS analysis was done using a 1 µL splitless injection on an Agilent HP-5MS capillary column (30 m × 0.25 mm inner diameter, 0.25 µm film thickness), according to the method described by Fiehn^65^. The same GC-MS system (Agilent 7890B coupled to a 5977B MSD, Agilent Technologies, USA) and analytical conditions (oven program, carrier gas, injection mode and MS parameters) as previously detailed in O’Connor et al. were used^65^. This method enables the quantification of desmosterol, lathosterol, lanosterol, campesterol, β-sitosterol, and cholestanol.

Desmosterol, lathosterol and lanosterol ratios to cholesterol are surrogate markers of cholesterol synthesis, while campesterol, β-sitosterol and cholestanol ratios to cholesterol indicate cholesterol absorption^45,64^. Finally, lathosterol to cholestanol ratio reflects the balance between cholesterol synthesis and absorption^66^.

### Subjective appetite sensations and appreciation of the diets

Perceived appetite sensations and appreciation of the experimental diets were assessed after seven and 28 days of intervention using visual analogue scales (VAS), as previously done in a similar population (i.e., French-Canadian adults) and settings (i.e., fully controlled feeding trial)^67^. Participants completed the VAS right before and immediately after each meal (breakfast, lunch and dinner) of the day, which was the same day for the 1-week and 4-week evaluation, in order to assess the same meals. VAS were lines of 150-mm and subjects were instructed to position a vertical bar on the scale to indicate how they felt in response to the four questions adapted from Hill & Blundell^68^:

1. How strong is your desire to eat? (very weak–very strong);
2. How hungry do you feel? (not hungry at all–as hungry as I have ever felt);
3. How full do you feel? (not full at all–very full);
4. How much food do you think you could eat? (nothing at all–a large amount).

These questions, respectively, assessed their perceived desire to eat, hunger, fullness, and prospective food consumption. Participants were also asked to complete a fifth VAS after eating to evaluate their appreciation of the meal they just consumed. A composite score was calculated from these four VAS to assess the general appetite sensation, hereafter referred to as the appetite score, using the following equation: (desire to eat + hunger + (150−fullness) + prospective food consumption)/4, as previously described^67,69,70^. In addition, the satiating capacity of the meal was evaluated using the satiety quotient, calculated as follows: ((post-meal fullness–pre-meal fullness)/(energy content of the test meal)) × 100^67,71^.

### Sample size calculation

The sample size for the intervention was determined to detect a 20% reduction in plasma LDL-C levels (primary outcome) following the WFPB diet compared with the SAD, with analyzes conducted separately by sex. Although we did not anticipate effect modification by sex, the trial was designed to ensure adequate power to detect clinically meaningful reductions in both males and females, thereby maximizing the potential for clinical translation, irrespective of sex. This expected effect size was a conservative estimate based on results from previous RCTs testing the impact of plant-rich diets on plasma lipids in individuals with non-familial hypercholesterolemia^13^. We assumed that the standard deviation of the within-subject difference in LDL-C levels between diets would be similar to the mean treatment effect^13,72^. Power calculations (GPower, v3.1.9.7) indicated that a sample size of 25 participants per sex would provide 80% power to detect such a difference at a two-sided alpha level of 5%. Input parameters for power calculations are presented in Supplementary Table 10^73^. Accordingly, we aimed to have 25 female and 25 male participants completing the trial. We originally accounted for an anticipated 20% dropout rate in our recruitment strategy, based on previous trials by our group^74^. As such, up to 62 participants (31 per sex) could be randomized to achieve 50 trial completers.

### Study outcomes

LDL-C was the primary outcome of the trial. The secondary outcome pertained to the dietary impact on the plasma metabolome and the metabolomic signature of the WFPB diet; this outcome is not reported in the current paper. All other outcomes reported in the current paper were exploratory. Among these exploratory outcomes, the following were prespecified: protocol adequacy (compliance, energy intake, and weight change during each diet, as well as weight and visceral adipose tissue mass at the end of each diet); other CVD risk factors; 10-year CVD risk; modification of the effect of diet on LDL-C according to sex, LDLR genotype, and diet sequence; and perceived appetite sensations and meal appreciation. Post hoc exploratory outcomes included effect modification by age; the proportion of participants whose post-diet LDL-C levels exceeded thresholds for FH investigation; plasma PCSK9; appetite hormones; non-cholesterol sterols; and correlates of the LDL-C and ApoB response to the WFPB diet.

### Statistical analysis

Statistical analysis was conducted using R version 4.3.0 within the R Studio 2023.06.1 environment. Descriptive statistics were calculated using the package *rstatix* (v0.7.2). All plots were created using the package *ggpubr* (v0.6.0). All statistical analyzes were two-sided with a significance threshold set at *P* < 0.05. We did not adjust *P* values or confidence intervals (CI) for multiple testing, as recommended by Schulz and Grimes^75^. Thus, results for exploratory outcomes should be interpreted with caution and considered as hypothesis-generating. Analyzes were first conducted using an intention-to-treat approach (*n* = 50). We also carried out per-protocol analyzes (*n* = 48) after excluding two participants with major protocol deviations (fully explained in the “Results”). There were no missing data in the datasets.

We first validated protocol adequacy by comparing compliance to each diet, mean daily energy intake during each diet, post-intervention weight and visceral adipose tissue mass, as well as weight change during each diet, using mixed models for repeated measures with the packages *lme4* (v1.1.35.5) and *lmerTest* (v3.1.3) with restricted maximum likelihood. Diet was treated as a fixed effect, while subjects were treated as a random effect (random intercept) to account for within-participant correlation, so that each participant served as their own control. The models were adjusted for BMI (kg/m^2^) and sequence (SAD-WFPB, WFPB-SAD).

To investigate the diet-induced change in LDL-C concentrations, along with other cardiovascular risk factors and estimated 10-year risk of CVD, we compared post-diet values using the same methodology. For these analyzes, the models were adjusted for BMI at the end of each diet (kg/m^2^), weight change during each intervention (%), and diet sequence (SAD-WFPB, WFPB-SAD). In these analyzes, the influence of each participant on the models was assessed by Cook’s distance using the package *influence.ME* (v0.9.9). If, for some outcomes, one or more participants had a high influence on the regression which led to a violation of the linear regression postulates, we repeated the analysis without this/these subjects. Normality was evaluated using the Shapiro–Wilk test and Q-Q plot with the packages *rstatix* (v0.7.2) and *stats* (v4.3.0), respectively. For outcomes with a non-normal residual distribution, a natural logarithm transformation was applied, and normality was reassessed. In such cases, unadjusted mean ± SEM were presented, and differences between diets were only presented as percentages and were estimated as 100 × exponential (mean difference of log values)−100.

In sensitivity analyzes, we tested whether unmodifiable risk factors, namely sex (female *vs* male), LDLR genotype (receptor-defective *vs* receptor-negative) and age (<40 years old *vs* ≥40 years old, as well as continuously), modified the effect of the WFPB, relative to the SAD, on LDL-C levels. The rationale for dichotomous age stratification is based on the Canadian Cardiovascular Society’s recommendation of different detection thresholds for untreated LDL-C levels in the Simplified Canadian Definition for FH (≥4.50 mmol/L for 18 to 40 years, and LDL-C>5.00 mmol/L for over 40 years)^76^. These analyzes were conducted by including their respective interaction terms in the models. These analyzes were then repeated on ApoB levels, in order to obtain a complementary perspective on the effect of diet on atherogenic lipoproteins, as ApoB is directly measured whereas LDL-C is calculated^77^. We also tested whether the diet sequence impacted the effect of the dietary interventions. Theses analyzes were carried out by adding an interaction between diet and diet sequence.

The following analyzes were intended to generate hypotheses. We used the per-protocol dataset (*n* = 48) to minimize potential confounding arising from protocol deviations. We first assessed whether diet could influence FH detection, given that none of the participants were using cholesterol-lowering medication during the dietary intervention, by comparing the proportion of participants whose post-diet LDL-C levels exceeded the thresholds for FH investigation defined in the Simplified Canadian Definition for FH^76^ (≥4.50 mmol/L for 18 to 40 years, and >5.00 mmol/L for over 40 years). Post-diet proportions were compared with McNemar’s test, implemented with the *rstatix* package (v0.7.2).

Second, the impact of the WFPB, relative to the SAD, on fasting appetite hormones (total GLP-1 and peptide YY), PCSK9 and noncholesterol sterols was assessed using linear mixed models, as described for LDL-C and CVD risk factors analyzes.

Third, we explored the correlates of the LDL-C response to the WFPB. Using the *stats* package (v4.3.0), we fitted a simple linear regression model including as potential correlates: unmodifiable factors (sex, age, LDLR genotype), lifestyle-related factors (BMI, as a surrogate of energy balance^78^, and mean calorie intake), cardiometabolic health surrogates (CRP, HOMA-IR, fasting total GLP-1, systolic blood pressure), and cholesterol and lipoprotein-metabolism related factors (lathosterol/cholesterol ratio, campesterol/cholesterol ratio, and levels of PCSK9, Lp(a) and LDL-C). For all potential correlates, we used values obtained at the end of the SAD phase to simulate the metabolic state of individuals before adopting a WFPB. Partial *R*^2^ values were calculated using the *rsq* package (v2.6). The LDL-C response was defined as the percent difference between post-WFPB and post-SAD levels and was treated as the dependent variable. Each potential correlate was treated as the independent variable. These analyzes were repeated on ApoB response.

Fourth, to support the translation of our findings into clinical practice, we compared (i) the impact of the diets on perceived appetite sensations and (ii) meal appreciation at the end of each diet, using linear mixed models. Diet was treated as a fixed effect, while subjects were treated as a random effect. We opted for an a priori sex-specific approach, motivated by findings from a previous study that reported marked differences between males and females in appetite sensations and diet appreciation following a Mediterranean diet^67^. This sex-specific analysis was intended to maximize the potential for clinical translation, regardless of sex. Thus, the models included an interaction term (diet * sex). In addition, the models were adjusted for meal (breakfast, lunch, and dinner), weekday (Monday, Tuesday, Wednesday, Thursday, Friday, Saturday, Sunday), daily energy intake (kcal), age (years), BMI (kg/m^2^), and sequence (SAD-WFPB, WFPB-SAD).

Finally, we evaluated changes in appetite sensations and meal appreciation between the first and last weeks of each dietary intervention to understand how repeated exposure to the diets influences these parameters and supports the translation of our findings into clinical practice. Again, an a priori sex-specific approach was used. For these analyzes, the assessment period (first week *vs* last week) was treated as a fixed effect, and participants were treated as a random effect. To determine the difference between the dietary interventions, an interaction term (assessment period * diet) was added to the models a priori. Two different models were fitted according to sex. To assess how sex impacts time-related changes between the two diets, linear mixed models were used. These models considered the changes in appetite sensations from the first to the final week of the interventions as the dependent variable. Additionally, an interaction term between sex and diet was included in the models to examine how the time-related effect of diet on appetite sensations might vary by sex. All these models were adjusted for meal (breakfast, lunch, and dinner), weekday (Monday, Tuesday, Wednesday, Thursday, Friday, Saturday, Sunday), daily energy intake (kcal), age (years), BMI (kg/m^2^), and sequence (SAD-WFPB, WFPB-SAD).

### Reporting summary

Further information on research design is available in the Nature Portfolio Reporting Summary linked to this article.

## Supplementary information

Supplementary Information

Reporting summary

Transparent Peer Review file

## Source data

Source data

## Data Availability

Source data are provided with this paper. All relevant data supporting the findings of this study are available within the main manuscript, the Supplementary Material, or in the Source Data file. Access to the minimum dataset necessary to interpret, verify, and extend the research reported in this article will be made available upon request through a custom proprietary repository, in accordance with the conditions set by the local ethics review committee on data sharing. Data access requests should be directed to the corresponding author. The study protocol, the CONSORT checklist, and the source data file are provided. Source data are provided with this paper.

## Code availability

Code is available via GitHub at https://github.com/Jacob-Lessard-Lord/FH_Diet_RCT_WFPB^79^.
