# Supplementary material for: Impact of a whole food, plant-based diet on LDL-cholesterol and cardiovascular risk factors in adults with heterozygous familial hypercholesterolemia: a randomized, two-period, two-treatment, crossover, fully controlled feeding trial
Source: Nat Commun. 2026 May 20;17:6632. doi: 10.1038/s41467-026-73468-4 (PMC13381664; doi:10.1038/s41467-026-73468-4)
Supplement: Supplementary file 1 — Supplementary Information [file 41467_2026_73468_MOESM1_ESM.pdf]

Impact of a whole food, plant-based diet on LDL-cholesterol and cardiovascular risk factors in adults with heterozygous familial hypercholesterolemia: a randomized, two-period, two-treatment, crossover, fully controlled feeding trial.

Supplemental material

Jacob Lessard-Lord, Valérie Guay, Maryka Rancourt-Bouchard, Patrick Couture, Anne Gangloff, Jonatan Blais, André J Tremblay, Karine Greffard, Jean-François Bilodeau, Iwona Rudkowska, Jean-Philippe Drouin-Chartier

Corresponding author:

Jean-Philippe Drouin-Chartier

jean-philippe.drouin-chartier@pha.ulaval.ca

Table of contents

Supplementary Table 1. Validation of the protocol adequacy, analyzed with intention to treat (n = 50) . 3

Supplementary Figure 1. Inter-individual variability in LDL-C (A) and ApoB (B) in the response to the WFPB relative to the SAD among the 50 study participants.. 4

Supplementary Table 2. Outcome levels at the end of the 4-week dietary interventions, analyzed per-protocol (n = 48) 5

Supplementary Table 3. Outcome levels at the end of the 4-week dietary interventions, analyzed with intention to treat, without influent subject[1] (n = 49) 6

Supplementary Table 4. Outcome levels at the end of the 4-week dietary interventions, analyzed per-protocol, without influent subject[1] (n = 47) 7

Supplementary Table 5. Effect of dietary intervention on LDL-C and ApoB after stratification by unmodifiable factors, analyzed per-protocol (n = 48).. 8

Supplementary Table 6. Effect of diet sequence on outcome levels at the end of the 4-week dietary interventions, analyzed with intention to treat (n = 50) 9

Supplementary Table 7. Changes in appetite sensation between the first week and the last week of each dietary intervention assessed with visual analog scales and stratified according to sex, analyzed per-protocol (n = 47). 10

Supplementary Figure 2. Differential response between females and males for appetite sensation difference between the first week and the last week of each dietary intervention, analyzed per-protocol (n = 47).. 12

Supplementary Figure 3. Appreciation of the meals after the two 4-week dietary interventions assessed with visual analog scales, analyzed per-protocol (n = 47)... 13

Supplementary Table 8. Correlates of the inter-individual variability of the diet-induced relative changes in LDL-C and ApoB levels (WFPB–SAD), analyzed per-protocol (n = 48). 14

Supplementary Note 1. Plant-Based Dietary Intervention Reporting Checklist.................................... 15

Supplementary Table 9. Overview of the 7-day rotating menu of each experimental diet.................... 17

Supplementary Image 1. Wednesday meals of SAD and WFPB menu. 19

Supplementary Table 10. Input parameters for the power calculation in GPower (v3.1.9.7)................ 20

Supplementary Table 1. Validation of the protocol adequacy, analyzed with intention to treat (n = 50)

Protocol adequacy	Mean ± SEM		WFPB–SAD difference, mean (95% CI)		*P* value
	SAD	WFPB	Absolute	Relative	
Compliance, %[1]	99.5 ± 0.1	99.2 ± 0.1	–0.3 (–0.6, 0.1)		0.11
Daily energy intake, kcal/day	2790 ± 51	2777 ± 51	–13 (–36, 9)	–0.5% (–1.3%, 0.3%)	0.24
Post–intervention weight, kg	76.4 ± 2	76.3 ± 2	–0.1 (–0.4, 0.2)	–0.1% (–0.5%, 0.3%)	0.45
Post-intervention visceral adipose tissue mass, g	624 ± 49	635 ± 49	11 (–30, 53)	1.8% (–4.8%, 8.5%)	0.58
Weight change during intervention, %[1,2]	–0.82 ± 0.22	–1.30 ± 0.22	–0.48 (–0.92, –0.05)		0.03

[1]For outcome with % as units, only absolute difference (in %) is presented to facilitate interpretation.
[2]Statistical significance was also assessed for each diet (P value $_{\text{SAD}}$ = 0.0004 and P value $_{\text{WFPB}}$ < 0.0001).
Statistical significance was assessed with linear mixed models adjusted for BMI (kg/m^2), and diet sequence (SAD-WFPB, WFPB-SAD), except compliance and post-intervention weight models were not adjusted for BMI. P values were obtained using two-sided test and were not adjusted for multiple comparison.

Supplementary Figure 1. Inter-individual variability in LDL-C (A) and ApoB (B) in the response to the WFPB relative to the SAD among the 50 study participants.
Each bar of the waterfall plots represents the change in LDL-C and ApoB of a single subject induced by the WFPB relative to the SAD. Each participant with an increase in LDL-C following WFPB compared with SAD was represented by a unique color to track within-subject concordance in LDL-C and ApoB responses.

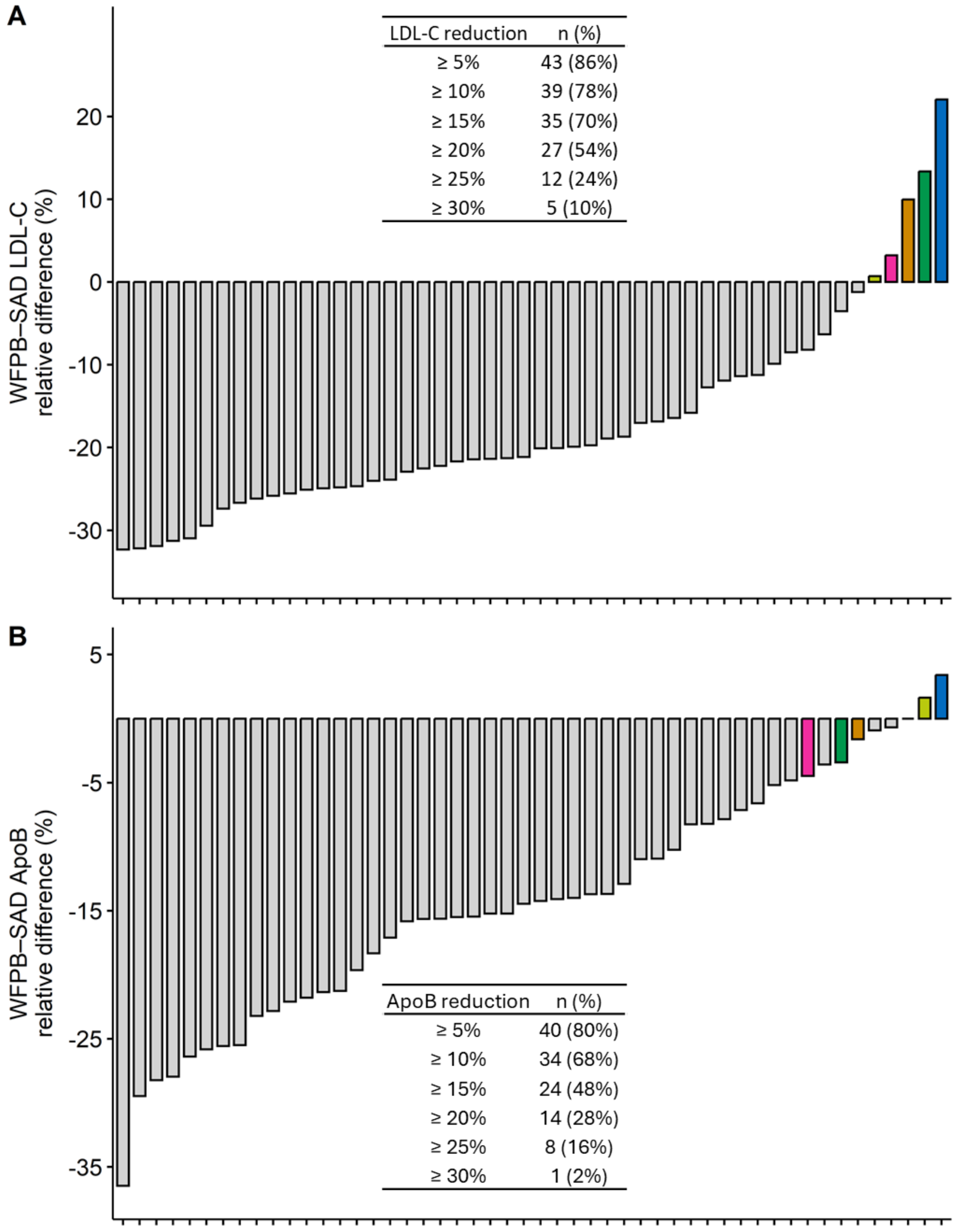

Supplementary Table 2. Outcome levels at the end of the 4-week dietary interventions, analyzed per-protocol (n = 48)

Outcomes	Mean ± SEM		WFPB–SAD difference, mean (95% CI)		*P* value
	SAD	WFPB	Absolute	Relative	
LDL-C, mmol/L (primary outcome)	7.21 ± 0.30	5.88 ± 0.30	–1.34 (–1.59, –1.08)	–18.6% (–22.1%, –15.0%)	<0.0001
Total cholesterol, mmol/L	9.21 ± 0.31	7.67 ± 0.31	–1.54 (–1.79, –1.28)	–16.7% (–19.4%, –13.9%)	<0.0001
LDL-$C_{Lp(a)\ corr.}$, mmol/L	7.07 ± 0.30	5.73 ± 0.30	–1.34 (–1.60, –1.09)	–19.0% (–22.6%, –15.4%)	<0.0001
HDL-C, mmol/L[1]	1.29 ± 0.05	1.17 ± 0.05	–0.12 (–0.17, –0.07)	–9.3% (–13.2%, –5.4%)	<0.0001
Triglycerides, mmol/L[1,2]	1.55 ± 0.14	1.34 ± 0.10	–	–8.8% (–14.6%, –2.5%)	0.008
Non-HDL-C, mmol/L	7.92 ± 0.31	6.50 ± 0.31	–1.42 (–1.66, –1.18)	–17.9% (–21.0%, –14.9%)	<0.0001
ApoA1, g/L	1.36 ± 0.03	1.26 ± 0.03	–0.11 (–0.14, –0.08)	–8.1% (–10.3%, –5.9%)	<0.0001
ApoB, g/L	1.9 ± 0.07	1.62 ± 0.07	–0.28 (–0.34, –0.22)	–14.7% (–17.9%, –11.6%)	<0.0001
Lp(a), nmol/L[1]	70.6 ± 11.0	72.3 ± 11.0	1.7 (–2.4, 5.8)	2.4% (–3.4%, 8.2%)	0.41
C-reactive protein, mg/L[2]	1.67 ± 0.28	1.58 ± 0.26	–	–6.0% (–22.2%, 13.7%)	0.52
Fasting glucose, mmol/L	5.02 ± 0.07	5.05 ± 0.07	0.03 (–0.07, 0.12)	0.6% (–1.4%, 2.4%)	0.55
Fasting insulin, pmol/L	53.1 ± 2.8	54.2 ± 2.8	1.1 (–3.2, 5.4)	2.1% (–6.0%, 10.2%)	0.61
HbA1c, %[3]	5.21 ± 0.04	5.17 ± 0.04	–0.04 (–0.08, 0.01)	–0.8% (–1.5%, 0.2%)	0.10
Blood pressure, mm Hg					
Systolic	112.4 ± 1.3	110.5 ± 1.3	–1.9 (–3.4, –0.3)	–1.7% (–3.0%, –0.3%)	0.02
Diastolic[1]	69.7 ± 1.2	67.3 ± 1.2	–2.4 (–4.1, –0.6)	–3.4% (–5.9%, –0.9%)	0.009
Ten-year risk of ASCVD event, %[2]	9.32 ± 1.44	7.62 ± 1.07	–	–11.7% (–19.5%, –3.1%)	0.01

[1]A subject had a high influence on the regression which led to a violation of the linear regression postulates (different subject for each outcome). Statistical analysis was re-done without this subject and presented in Table S5. Nevertheless, this violation of the regression postulates did not change the statistical significance of the results.

[2]Analyses were performed on log-transformed data. In such cases, unadjusted mean ± SEM are presented and differences between diets are only presented as percentages and were estimated as 100 × exponential (mean difference of log values) − 100.

Statistical significance was assessed with linear mixed models adjusted for BMI at the end of each diet (kg/m^2), weight change during each intervention (%), and diet sequence (SAD-WFPB, WFPB-SAD). P values were obtained using two-sided test and were not adjusted for multiple comparison.

Supplementary Table 3. Outcome levels at the end of the 4-week dietary interventions, analyzed with intention to treat, without influent subject[1] (n = 49)

Outcomes	Mean ± SEM		WFPB–SAD difference, mean (95% CI)		*P* value
	SAD	WFPB	Absolute	Relative	
HDL-C, mmol/L	1.28 ± 0.04	1.18 ± 0.04	–0.10 (–0.14, –0.06)	–7.8% (–10.9%, –4.7%)	<0.0001
Triglycerides, mmol/L[2]	1.48 ± 0.12	1.31 ± 0.10	–	–8.6% (–13.8%, –3.0%)	0.004
Lp(a), nmol/L	69.7 ± 12.0	72.7 ± 12.0	3.0 (–0.3, 6.3)	4.3% (–0.4%, 9.0%)	0.07
Diastolic blood pressure, mm Hg	68.8 ± 1.1	67.0 ± 1.1	–1.8 (–3.2, –0.4)	–2.6% (–4.7%, –0.6%)	0.01

[1]Determined using Cook's distance.

[2]Analyses were performed on log-transformed data. In such cases, unadjusted mean ± SEM are presented and differences between diets are only presented as percentages and were estimated as 100 × exponential (mean difference of log values) − 100.

Statistical significance was assessed with linear mixed models adjusted for BMI at the end of each diet (kg/m^2), weight change during each intervention (%), and diet sequence (SAD-WFPB, WFPB-SAD). Ten-year risk ASCVD model was not adjusted for age and sex, since these parameters were used to calculate this outcome. P values were obtained using two-sided test and were not adjusted for multiple comparison.

Supplementary Table 4. Outcome levels at the end of the 4-week dietary interventions, analyzed per-protocol, without influent subject[1] (n = 47)

Outcomes	Mean ± SEM		WFPB–SAD difference, mean (95% CI)		*P* value
	SAD	WFPB	Absolute	Relative	
HDL-C, mmol/L	1.28 ± 0.05	1.18 ± 0.05	–0.10 (–0.14, –0.06)	–7.8% (–10.9%, –4.7%)	<0.0001
Triglycerides, mmol/L[2]	1.49 ± 0.13	1.34 ± 0.11	–	–7.8% (–13.0%, –2.2%)	0.008
Lp(a), nmol/L	63.0 ± 9.1	66.0 ± 9.1	3.1 (–0.3, 6.5)	4.9% (–0.5%, 10.3%)	0.08
Diastolic blood pressure, mm Hg	69.0 ± 1.1	67.3 ± 1.1	–1.8 (–3.2, –0.4)	–2.6% (–4.6%, –0.6%)	0.01

[1]Determined using Cook's distance.

[2]Analyses were performed on log-transformed data. In such cases, unadjusted mean ± SEM are presented and differences between diets are only presented as percentages and were estimated as 100 × exponential (mean difference of log values) − 100.

Statistical significance was assessed with linear mixed models adjusted for BMI at the end of each diet (kg/m^2), weight change during each intervention (%), and diet sequence (SAD-WFPB, WFPB-SAD). Ten-year risk ASCVD model was not adjusted for age and sex, since these parameters were used to calculate this outcome. P values were obtained using two-sided test and were not adjusted for multiple comparison.

Supplementary Table 5. Effect of dietary intervention on LDL-C and ApoB after stratification by unmodifiable factors, analyzed per-protocol (n = 48).

Stratification	Stratification level	n	LDL-C				ApoB			
			WFPB–SAD relative difference, mean (95% CI)	P value	P value for interaction		WFPB–SAD relative difference, mean (95% CI)	P value	P value for interaction	
					Stratified	Continuous			Stratified	Continuous
Sex[1]										
	Female	25	–17.6% (–22.1%, –13.2%)	<0.0001	0.90	---	–15.4% (–19.3%, –11.5%)	<0.0001	0.42	---
	Male	23	–19.7% (–25.1%, –14.4%)	<0.0001			–14.1% (–18.8%, –9.4%)	<0.0001		
LDLR genotype[1]										
	Receptor–defective	14	–18.7% (–24.6%, –12.8%)	<0.0001	0.53	---	–14.2% (–19.6%, –8.9%)	<0.0001	0.93	---
	Receptor–negative	30	–18.0% (–22.5%, –13.4%)	<0.0001			–14.9% (–18.9%, –10.9%)	<0.0001		
Age										
	< 40 years old	25	–19.3% (–24.4%, –14.1%)	<0.0001	0.83	0.31	–15.2% (–19.7%, –10.6%)	<0.0001	0.74	0.35
	≥ 40 years old	23	–17.9% (–22.4%, –13.3%)	<0.0001			–14.6% (–18.6%, –10.6%)	<0.0001		

[1]These stratified analyses were pre-specified for LDL-C

Statistical significance was assessed with linear mixed models adjusted for BMI at the end of each diet (kg/m^2), weight change during each intervention (%), and diet sequence (SAD-WFPB, WFPB-SAD). Models also included the stratification variable and the interaction term between LDL-C/ApoB and the stratification variable. P values were obtained using two-sided test and were not adjusted for multiple comparison.

Supplementary Table 6. Effect of diet sequence on outcome levels at the end of the 4-week dietary interventions, analyzed with intention to treat (n = 50)

Outcomes	SAD–WFPB (n = 27)		WFPB–SAD (n = 23)		*P* value for sequence interaction
	WFPB–SAD relative difference mean (95% CI)	*P* value	WFPB–SAD relative difference mean (95% CI)	*P* value	
LDL-C, mmol/L (primary outcome)	–17.3% (–22.2%, –12.3%)	<0.0001	–18.7% (–23.8%, –13.6%)	<0.0001	0.72
Total cholesterol, mmol/L	–15.5% (–19.4%, –11.5%)	<0.0001	–16.9% (–20.9%, –12.8%)	<0.0001	0.67
LDL-$C_{Lp(a)\ corr.}$, mmol/L	–17.7% (–22.7%, –12.7%)	<0.0001	–19.1% (–24.2%, –14.0%)	<0.0001	0.72
HDL-C, mmol/L	–6.9% (–12.2%, –1.7%)	0.01	–10.1% (–15.4%, –4.9%)	0.0005	0.39
Triglycerides, mmol/L[1]	–11.3% (–18.9%, –3.1%)	0.01	–7.6% (–15.6%, 1.2%)	0.10	0.52
Non-HDL-C, mmol/L	–16.9% (–21.2%, –12.6%)	<0.0001	–18.0% (–22.4%, –13.5%)	<0.0001	0.78
ApoA1, g/L	–7.0% (–10.2%, –3.7%)	0.0001	–8.4% (–11.8%, –5.0%)	<0.0001	0.55
ApoB, g/L	–14.1% (–18.3%, –9.8%)	<0.0001	–15.2% (–19.4%, –10.9%)	<0.0001	0.67
Lp(a), nmol/L	1.9% (–4.5%, 8.3%)	0.58	2.5% (–5.4%, 10.4%)	0.54	0.97
C-reactive protein, mg/L[1]	19.1% (–8.3%, 55.5%)	0.20	–20.9% (–39.6%, 3.6%)	0.10	0.04
Fasting glucose, mmol/L	–0.9% (–3.3%, 1.5%)	0.48	1.9% (–0.7%, 4.5%)	0.15	0.13
Fasting insulin, mg/L	9.0% (–1.2%, 19.4%)	0.09	–4.3% (–14.8%, 6.3%)	0.44	0.08
HbA1c, %	–1.1% (–2.2%, –0.1%)	0.04	0.0% (–1.1%, 1.1%)	0.98	0.15
Blood pressure, mm Hg					
Systolic	–1.4% (–3.2%, 0.3%)	0.13	–1.7% (–3.5%, 0.2%)	0.08	0.85
Diastolic	–2.6% (–5.9%, 0.7%)	0.13	–4.3% (–7.8%, –0.8%)	0.02	0.50
Ten-year risk of ASCVD event, %[1]	–15.1% (–24.8%, –4.3%)	0.01	–8.3% (–18.9%, 3.6%)	0.17	0.39

[1]Analyses were performed on log-transformed data. In such cases, unadjusted mean ± SEM are presented and differences between diets are only presented as percentages and were estimated as 100 × exponential (mean difference of log values) − 100.
Statistical significance was assessed with linear mixed models adjusted for BMI at the end of each diet (kg/m^2), weight change during each intervention (%), and diet sequence (SAD-WFPB, WFPB-SAD). Models also included the interaction term between the outcome and the diet sequence. P values were obtained using two-sided test and were not adjusted for multiple comparison.

Supplementary Table 7. Changes in appetite sensation between the first week and the last week of each dietary intervention assessed with visual analog scales and stratified according to sex, analyzed per-protocol (n = 47)[1].

Appetite sensations	SAD		WFPB		*P* value for between diet interaction
	Week 4–Week 1 relative difference mean (95% CI)	*P* value	Week 4–Week 1 relative difference mean (95% CI)	*P* value	
Females (n = 25)					
Appetite ratings before meals					
Desire to eat	–5.3% (–16.7%, 6.3%)	0.38	3.9% (–9.6%, 17.4%)	0.58	0.31
Hunger	–4.4% (–16.4%, 7.8%)	0.49	11.1% (–3.5%, 25.8%)	0.14	0.13
Fullness	11.5% (–9.8%, 32.5%)	0.29	–3.4% (–18.6%, 11.9%)	0.67	0.29
Prospective food consumption	–2.7% (–13.1%, 7.9%)	0.62	1.0% (–9.8%, 11.9%)	0.85	0.63
Appetite score	–4.1% (–13.4%, 5.3%)	0.40	4.6% (–5.8%, 15.1%)	0.39	0.23
Appetite ratings after meals					
Desire to eat[2]	–30.6% (–50.9%, –3.4%)	0.04	28.0% (–7.3%, 78.4%)	0.14	0.01
Hunger[2]	–11.0% (–36.6%, 23.1%)	0.50	27.0% (–7.4%, 75.7%)	0.15	0.14
Fullness	2.2% (–6.1%, 10.5%)	0.61	3.6% (–3.7%, 10.7%)	0.34	0.76
Prospective food consumption[2]	–15.2% (–38.7%, 15.9%)	0.32	21.5% (–10.2%, 66.1%)	0.22	0.12
Appetite score[2]	–15.5% (–32.9%, 5.5%)	0.15	16.1% (–6.3%, 44.8%)	0.19	0.05
Satiety quotient	–5.1% (–24.4%, 14.5%)	0.62	9.3% (–10.5%, 28.6%)	0.36	0.32
Meal appreciation	–2.6% (–13.3%, 8.4%)	0.64	–6.1% (–17.0%, 4.7%)	0.28	0.68
Males (n = 22)					
Appetite ratings before meals					
Desire to eat	5.2% (–3.1%, 13.2%)	0.22	–0.3% (–9.1%, 8.5%)	0.95	0.35
Hunger	3.6% (–5.7%, 12.6%)	0.44	–2.9% (–12.8%, 7.0%)	0.58	0.35
Fullness	–6.5% (–25.6%, 12.9%)	0.51	8.6% (–8.2%, 25.4%)	0.32	0.24
Prospective food consumption	4.1% (–3.9%, 11.7%)	0.31	2.7% (–5.6%, 10.8%)	0.53	0.77
Appetite score	3.6% (–3.6%, 10.6%)	0.33	–0.9% (–8.5%, 6.7%)	0.82	0.39
Appetite ratings after meals					
Desire to eat[2]	53.0% (8.6%, 113.2%)	0.02	3.6% (–25.2%, 43.6%)	0.83	0.11
Hunger[2]	34.1% (–4.1%, 85.7%)	0.09	7.9% (–21.9%, 49.3%)	0.65	0.36
Fullness	–5.3% (–12.1%, 1.7%)	0.14	–2.2% (–8.5%, 4.1%)	0.50	0.56
Prospective food consumption[2]	21.1% (–10.7%, 62.9%)	0.22	28.5% (–3.8%, 72.4%)	0.10	0.78
Appetite score[2]	28.4% (1.2%, 61.7%)	0.04	9.2% (–13.0%, 37.6%)	0.46	0.34
Satiety quotient	–3.9% (–20.5%, 12.6%)	0.65	–9.5% (–25.1%, 6.2%)	0.24	0.63
Meal appreciation	–3.6% (–10.3%, 2.7%)	0.28	5.0% (–2.0%, 11.8%)	0.17	0.08

Statistical significance was assessed with linear mixed models adjusted for meal (breakfast, lunch, and dinner), weekday (Monday, Tuesday, Wednesday, Thursday, Friday, Saturday, Sunday), daily energy intake (kcal), BMI (kg/m^2), and diet sequence (SAD-WFPB, WFPB-SAD). P values were obtained using two-sided test and were not adjusted for multiple comparison.
[1]A subject did not complete the visual analog scales.
[2]Analyses were performed on log-transformed data. In such cases, differences between diets are presented as percentages and were estimated as 100 × exponential (mean difference of log values) – 100.

Supplementary Figure 2. Differential response between females and males for appetite sensation difference between the first week and the last week of each dietary intervention, analyzed per-protocol (n = 47). Results are expressed as mean (points) and SEM (lines). Statistical significance was assessed by linear mixed models including the interaction between dietary intervention (SAD, WFPB) and sex (female, male) and adjusted for meal (breakfast, lunch, and dinner), differential daily energy intake between the first and the last week of each dietary intervention (kcal), BMI at the end of the intervention (kg/m^2), and diet sequence (SAD-WFPB, WFPB-SAD). P values were obtained using two-sided test and were not adjusted for multiple comparison.

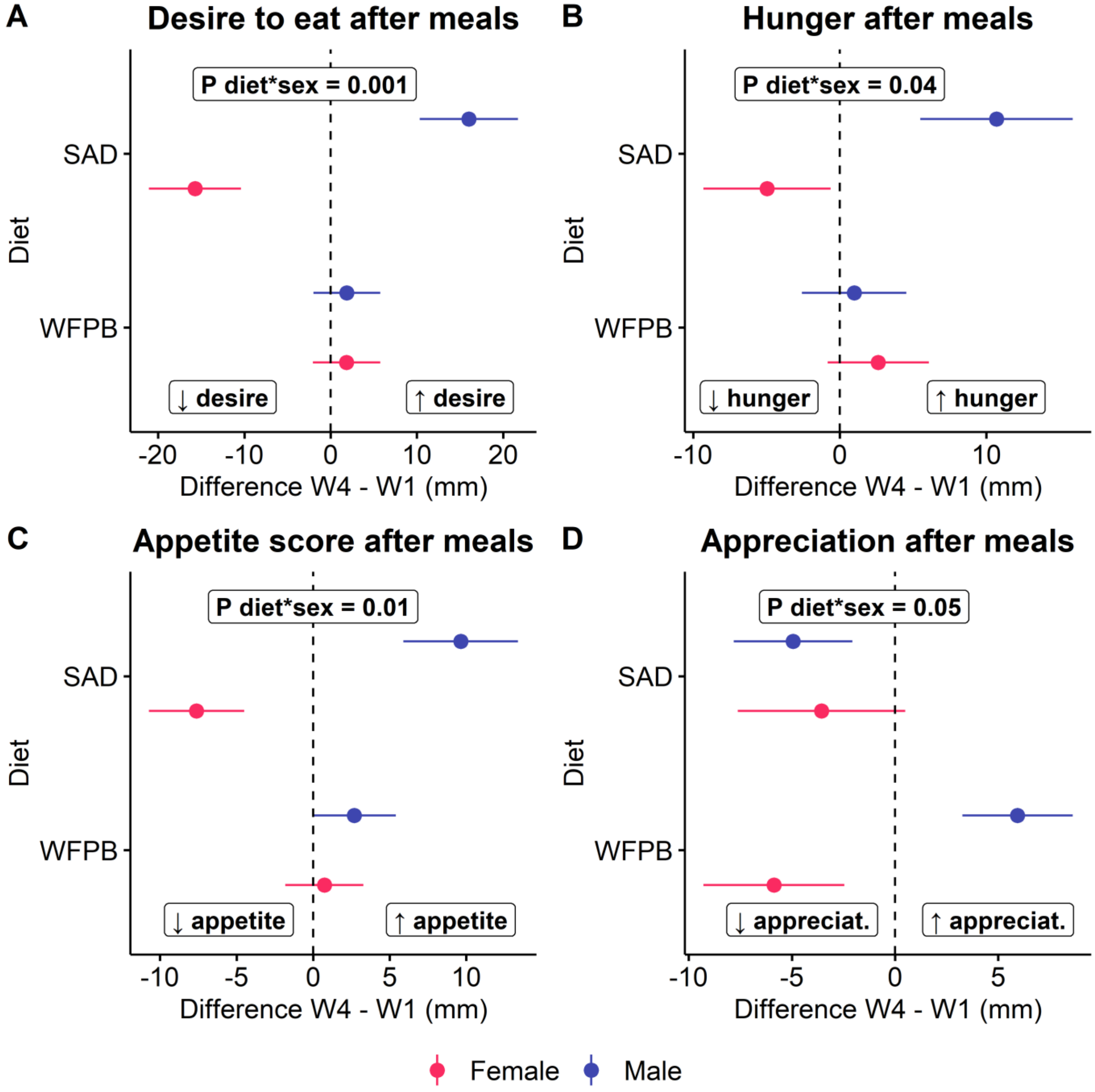

Supplementary Figure 3. Appreciation of the meals after the two 4-week dietary interventions assessed with visual analog scales, analyzed per-protocol (n = 47). Each point represents the appreciation of a single meal (breakfast, lunch, and dinner) by a single subject. Appreciation is expressed as length measured (in mm) on visual analog scales, with higher length representing higher meal appreciation.

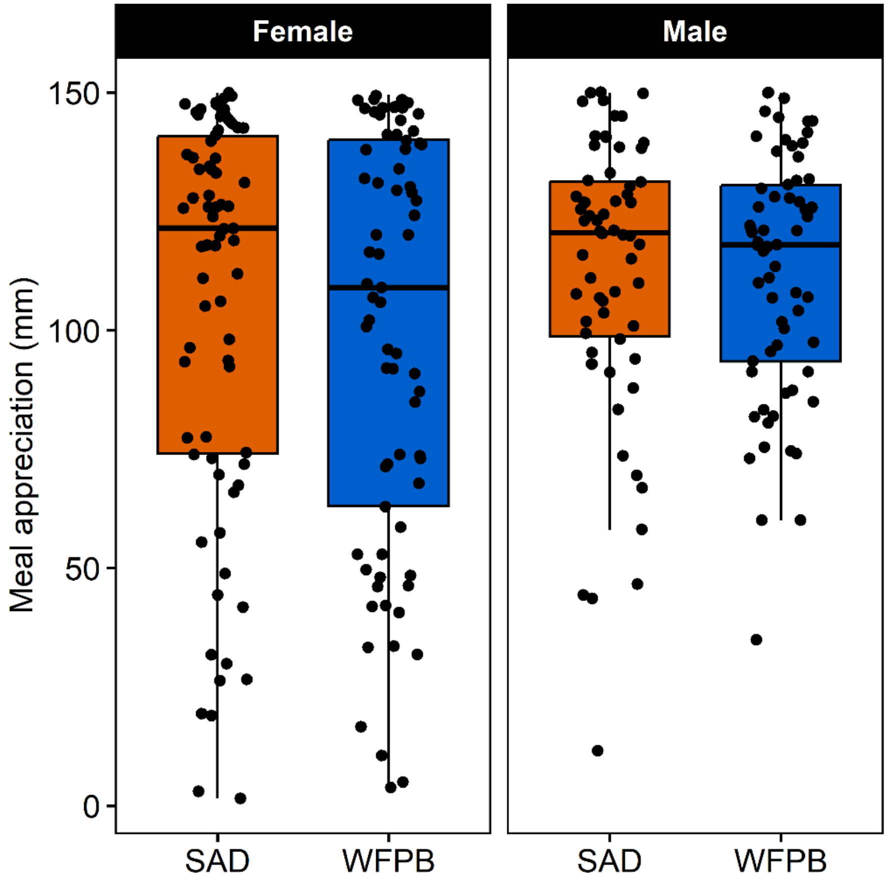

Supplementary Table 8. Correlates of the inter-individual variability of the diet-induced relative changes in LDL-C and ApoB levels (WFPB–SAD), analyzed per-protocol (n = 48).

Independent variable	β (95% CI)	Partial R^2, %	*P* value
LDL-C relative change WFPB-SAD (%)			
Sex (female vs male)	–0.28 (–16.90, 16.34)	0.0	0.97
Age (years)	0.15 (–0.24, 0.55)	2.0	0.43
LDLR genotype (RD vs RN)	0.80 (–8.30, 9.90)	3.1	0.86
BMI (per kg/m^2)	0.12 (–1.11, 1.34)	0.1	0.85
LDL–C level (per mmol/L)	–1.82 (–3.91, 0.28)	9.2	0.09
Lp(a) level (per 10 nmol/L)	–0.14 (–0.67, 0.40)	0.9	0.61
PCSK9 level (per ng/mL)	0.01 (–0.02, 0.04)	1.4	0.50
C–reactive protein (per mg/L)	1.69 (–0.88, 4.26)	5.5	0.19
HOMA-IR (per point)	1.02 (–4.05, 6.09)	2.6	0.68
Fasting total GLP-1 (per pmol/L)	–0.04 (–0.24, 0.17)	0.4	0.72
Systolic blood pressure (per mm Hg)	–0.16 (–0.76, 0.44)	0.9	0.60
Lathosterol/cholesterol (per 100 µmol/mmol)	–0.09 (–0.26, 0.08)	3.4	0.31
Campesterol/cholesterol (per 100 µmol/mmol)	–0.01 (–0.14, 0.12)	0.0	0.91
Mean calorie intake (per 100 kcal)	0.35 (–1.94, 2.64)	0.3	0.76
Model[1]		24.5 (–12.0)	0.79
ApoB relative change WFPB-SAD (%)			
Sex (female vs male)	4.18 (–8.10, 16.46)	1.5	0.49
Age (years)	0.07 (–0.23, 0.36)	0.7	0.65
LDLR genotype (RD vs RN)	–1.93 (–8.66, 4.79)	2.2	0.56
BMI (per kg/m2)	–0.43 (–1.33, 0.47)	3.0	0.34
ApoB level (per g/L)	–6.25 (–13.61, 1.12)	8.8	0.09
Lp(a) level (per 10 nmol/L)	–0.15 (–0.54, 0.25)	1.8	0.46
PCSK9 level (per ng/mL)	0.01 (–0.01, 0.03)	1.4	0.51
C–reactive protein (per mg/L)	1.62 (–0.28, 3.51)	8.9	0.09
HOMA-IR (per point)	1.92 (–1.87, 5.72)	3.4	0.31
Fasting total GLP-1 (per pmol/L)	–0.15 (–0.30, –0.01)	12.6	0.04
Systolic blood pressure (per mm Hg)	–0.22 (–0.67, 0.22)	3.3	0.32
Lathosterol/cholesterol (per 100 µmol/mmol)	–0.05 (–0.17, 0.08)	1.8	0.46
Campesterol/cholesterol (per 100 µmol/mmol)	–0.06 (–0.16, 0.03)	5.1	0.20
Mean calorie intake (per 100 kcal)	0.47 (–1.21, 2.15)	1.0	0.57
Model[1]		36.6 (5.9)	0.33

[1]R^2 of the model (adjusted R^2).

Statistical significance was assessed with linear models. For this analysis, post-SAD levels were used for all independent variables. P values were obtained using two-sided test and were not adjusted for multiple comparison.

Supplementary Note 1. Plant-Based Dietary Intervention Reporting Checklist
Checklist: Storz, M.A. What makes a plant-based diet? a review of current concepts and proposal for a standardized plant-based dietary intervention checklist. *Eur J Clin Nutr* 76, 789–800 (2022). https://doi.org/10.1038/s41430-021-01023-z

1. **The plant-based diet in the present study included:**

☒ Meat ☒ Poultry ☒ Fish/Seafood ☒ Dairy ☒ Eggs ☒ Honey

2. **If animal products were included at what frequency? Please specify:**
   - Meat: 1x per week
   - Poultry: 3x per week
   - Fish/Seafood: 3x per week
   - Dairy: 5x per week
   - Eggs: 2x per week
   - Honey: 3x per week

3. **Please describe a potential "whole-foods aspect". The dietary intervention explicitly:**

☒ **favored** whole/unprocessed foods;
☒ **restricted**: ☒ processed foods ☒ added sugars ☒ added oils

4. **Did the intervention restrict calories or portion sizes?**

☐ No, *ad-libitum* calorie intake;
☒ Yes, total calorie intake / portion size restricted: to avoid any weight change during the dietary intervention.

5. **Did the intervention restrict sodium intake?**

☐ No, *ad-libitum* sodium intake;
☒ Yes, daily sodium intake was restricted to: the sodium in the provided diet (mean ± SD: 2519 ± 285 mg/2500kcal/day).

6. **Did the intervention restrict intake of "fatty" plant-foods?**

☐ No *ad-libitum* intake;
☒ Yes, the intervention restricted: ☒ nuts and seeds (mean ± SD: 40 ± 20 g/2500 kcal/day) ☒ oils (35% of daily energy intake from lipids) ☒ avocados (no avocado was provided to the participants)

7. **Was there a target for macronutrient distribution?**

☐ No;
☒ Yes, please specify: Daily energy intake was 50% from carbohydrates, 15% from protein, and 35% from fat.

8. **Were participants asked to take any specific supplements during the dietary intervention?**

☒ No;
☐ Yes, ☐ Vitamin B12 ☐ Vitamin D ☐ Calcium ☐ Iodine ☐ Others

9. **Did participants receive any kind of support or supervision during the dietary modification?**

☐ No;
☒ Yes
- ☒ written instructions
- ☐ classes and group sessions
- ☐ cooking demonstrations
- ☒ others Please specify: All meals and beverages were provided to the participants.

Supplementary Table 9. Overview of the 7-day rotating menu of each experimental diet.

	Monday		Tuesday		Wednesday		Thursday		Friday		Saturday		Sunday	
	SAD	WFPB	SAD	WFPB	SAD	WFPB	SAD	WFPB	SAD	WFPB	SAD	WFPB	SAD	WFPB
**Breakfast**	Sesame seeds bagel	Whole wheat bagel	Oat bread	Oat bread	Commercial cereals (apple and cinnamon)	Overnight oats with apple and cinnamon	Sesame seeds bagel	Whole wheat bagel	English muffins with eggs, cheese, and ham	Whole wheat burritos	Oat bread	Oat bread	Waffles with maple syrup	Whole wheat pancakes with maple syrup
	Cream cheese	Low-fat cheddar cheese	Peanut butter	Almond butter	Milk		Peanut butter	Almond butter		Cheese	Pork spread and butter	Vegetarian pâté	Vanilla yogurt	Plain soy yogurt
	Orange	Orange	Milk	Milk	Red grape	Red grape	Strawberry and blueberry smoothie	Strawberry and blueberry smoothie	Orange	Orange	Red grape	Orange juice	Tropical fruit salad	Fruit salad
**Snack**	Banana bread	Oat granola bars	Fruit salad and social tea biscuits	Fruit salad	Date cookie	Bran and raisin muffins	Chocolate milk	Green grape and chocolate soy drink	Nut trail mix	Roasted almonds	Chocolate milk	Red grape and vanilla soy drink	Zucchini muffin	Zucchini and nut muffins
**Lunch**	Beef chili	Vegetarian chili	Chinese-style rice with vegetables and chicken	Shrimp rice salad	Curry chicken	Vegetable couscous with chickpea	Vegetable soup	Vegetable and lentil soup	Vegetable juice	Squash soup	Veggies and hummus	Veggies and hummus	Pea soup	White bean soup
	Corn chips	Multigrain pita chips		Veggies and dip	Roasted vegetables		Ham and cheese sandwich	Tuna salad with grilled tortillas	Shepherd's pie with beef	Shepherd's pie with turkey and lentil	Chicken pot pie	Greek chicken pita	Cheese and vegetable omelette	Cheese and vegetable frittata
	Low-fat cheddar cheese	Low-fat cheddar cheese			Couscous		Pretzels	Low-fat cheddar cheese	Ketchup	Ketchup			Oat bread	Orange
	Brownies	Date brownies bites	Chocolate chip cookies	Apple	Strawberry yogurt	Greek yogurt and berries	Apple	Apple	Strawberry yogurt	Silken tofu and peach verrine	Applesauce and social tea biscuits	Fruit salad	Chocolate chunks	Oat bread with dark chocolate chunks
**Dinner**	Lemon chicken	Tofu and vegetable stir-fry	Meatballs with Swedish sauce	Italian-style veal meatballs	Spaghetti with meat sauce	Vegetarian spaghetti	BBQ chicken	BBQ chicken	Deluxe pizza	Vegetable pizza	Salmon pasta	Honey salmon	Jambalaya (chicken and sausage)	Jambalaya (vegetarian)
	Bell peppers	Whole wheat pasta	Mashed potatoes	Vegetable quinoa	Caesar salad	Garden salad	Herb-seasoned potatoes	Herb-seasoned potatoes	Chips	Roasted chickpea snacks		Whole wheat pasta		Cherry tomato salads
	Garlic pasta	Roasted nuts	Broccoli and cauliflower	Broccoli and cauliflower		Parmesan vegetalian substitute	Coleslaw	Coleslaw				Green beans		
	Almond cake	Cantaloupe	Vanilla yogurt	Silken tofu and mango verrine	Peanut brittle	Dates and walnuts	Lemon poppy seed bread	Cantaloupe	Chocolate cake	Apple	Apple dessert	Apple and blueberry crisp	Carrot cake	Cantaloupe

**Beverage**	Apple and grape juice	Water		Apple cocktail	Water		Orange juice	Water		Cranberry cocktail	Water		Apple and grape juice	Water		Apple cocktail	Water		Orange juice	Water

Homemade recipes are highlighted in blue (SAD: n=28; WFPB: n=47). All WFPB recipes are available at https://github.com/Jacob-Lessard-Lord/FH_Diet_RCT_WFPB.

Supplementary Image 1. Wednesday meals of SAD and WFPB menu. The photos show the portion sizes corresponding to a daily energy intake of 2500 kcal.

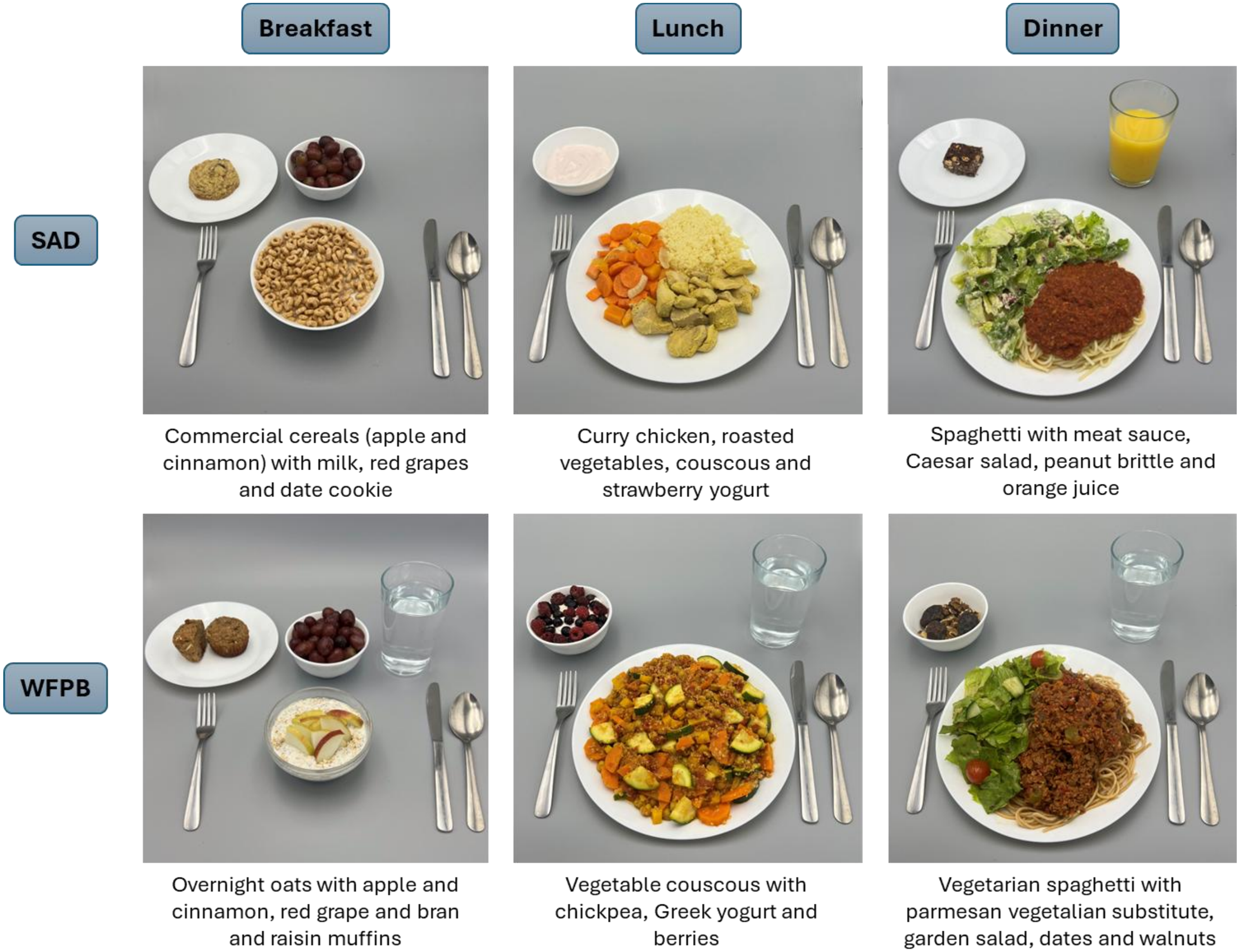

Supplementary Table 10. Input parameters for the power calculation in GPower (v3.1.9.7).

Parameter	Value
Test family	F tests
Statistical test	ANOVA: Repeated measures, within factors
Effect size f	0.30
Alpha	0.05
Power	0.80
Number of groups (diets)	2
Number of measurements	2
Correlation among repeated measures	0.5
Nonsphericity correction	1
