# Supplementary material for: Impact of a whole food, plant-based diet on LDL-cholesterol and cardiovascular risk factors in adults with heterozygous familial hypercholesterolemia: a randomized, two-period, two-treatment, crossover, fully controlled feeding trial
Source: Nat Commun. 2026 May 20;17:6632. doi: 10.1038/s41467-026-73468-4 (PMC13381664; doi:10.1038/s41467-026-73468-4)
Supplement: Supplementary file 2 — Reporting summary [file 41467_2026_73468_MOESM2_ESM.pdf]

nature portfolio

Corresponding author(s): Jean-Philippe Drouin-Chartier

Last updated by author(s): Mar 16, 2026

Reporting Summary

Nature Portfolio wishes to improve the reproducibility of the work that we publish. This form provides structure for consistency and transparency in reporting. For further information on Nature Portfolio policies, see our Editorial Policies and the Editorial Policy Checklist.

Statistics

For all statistical analyses, confirm that the following items are present in the figure legend, table legend, main text, or Methods section.

n/a	Confirmed	
☐	☒	The exact sample size (*n*) for each experimental group/condition, given as a discrete number and unit of measurement
☒	☐	A statement on whether measurements were taken from distinct samples or whether the same sample was measured repeatedly
☐	☒	The statistical test(s) used AND whether they are one- or two-sided<br>*Only common tests should be described solely by name; describe more complex techniques in the Methods section.*
☐	☒	A description of all covariates tested
☐	☒	A description of any assumptions or corrections, such as tests of normality and adjustment for multiple comparisons
☐	☒	A full description of the statistical parameters including central tendency (e.g. means) or other basic estimates (e.g. regression coefficient) AND variation (e.g. standard deviation) or associated estimates of uncertainty (e.g. confidence intervals)
☐	☒	For null hypothesis testing, the test statistic (e.g. *F*, *t*, *r*) with confidence intervals, effect sizes, degrees of freedom and *P* value noted<br>*Give P values as exact values whenever suitable.*
☒	☐	For Bayesian analysis, information on the choice of priors and Markov chain Monte Carlo settings
☒	☐	For hierarchical and complex designs, identification of the appropriate level for tests and full reporting of outcomes
☒	☐	Estimates of effect sizes (e.g. Cohen's *d*, Pearson's *r*), indicating how they were calculated

Our web collection on statistics for biologists contains articles on many of the points above.

Software and code

Policy information about availability of computer code

Data collection	No software was used for data collection.
Data analysis	Statistical analysis was conducted using R version 4.3.0 within the R Studio 2023.06.1 environment. Descriptive statistics were calculated using the package rstatix (v0.7.2). All plots were created using the package ggpubr (v0.6.0). The packages lme4 (v1.1.35.5), lmerTest (v3.1.3) and influence.ME (v0.9.9)were used for mixed models for repeated measures. For the linear regression model, olsrr package (v0.6.0) and rsq package (v2.6) were used.<br><br>Code is available via GitHub at https://github.com/Jacob-Lessard-Lord/FH_Diet_RCT_WFPB.

For manuscripts utilizing custom algorithms or software that are central to the research but not yet described in published literature, software must be made available to editors and reviewers. We strongly encourage code deposition in a community repository (e.g. GitHub). See the Nature Portfolio guidelines for submitting code & software for further information.

Data

Policy information about availability of data

All manuscripts must include a data availability statement. This statement should provide the following information, where applicable:

- Accession codes, unique identifiers, or web links for publicly available datasets
- A description of any restrictions on data availability
- For clinical datasets or third party data, please ensure that the statement adheres to our policy

Source data are provided with this paper. All relevant data supporting the findings of this study are available within the main manuscript, the Supplementary Material, or in the Source Data file. Access to the minimum dataset necessary to interpret, verify, and extend the research reported in this article will be made available upon request through a custom proprietary repository, in accordance with the conditions set by the local ethics review committee on data sharing. Data access requests should be directed to the corresponding author. The study protocol, the CONSORT checklist, and the source data file are provided.

Research involving human participants, their data, or biological material

Policy information about studies with human participants or human data. See also policy information about sex, gender (identity/presentation), and sexual orientation and race, ethnicity and racism.

Reporting on sex and gender	In this study, sex was self-reported and used a biological variable. We conducted sex-stratified analyses on LDL-C, ApoB and appetite sensations. Sex was used as a covariate in all statistical analyses.
Reporting on race, ethnicity, or other socially relevant groupings	All subjects were Caucasian (French-Canadian) living in Quebec City.
Population characteristics	Males (n=24) and females (n=26), with genetically confirmed heterozygous familial hypercholesterolemia (HeFH) between the age of 19 and 58 years old participated to the study.
Recruitment	Volunteers were recruited through referral from physician collaborators at the Lipid Clinic of the CHU de Québec-Université Laval in Quebec City, Canada. To be eligible, participants had to be adults (18 to 60 years old) with genetically confirmed HeFH. Data on the FH-causing variant and LDLR genotype were obtained from medical records. Premenopausal female participants were required to have a regular menstrual cycle for more than 3 months and postmenopausal female participants were required to not use hormone replacement therapy. Exclusion criteria included any cardiovascular disease-risk exacerbating conditions (e.g., over 60 years old, homozygous FH, personal history of cardiovascular disease, diabetes/anti-diabetic drug use, severe obesity, unstable body weight for more than 3 months, uncontrolled hypertension and genetic hypertriglyceridemia), allergies or aversions to components of the experimental diets and any conditions that would interfere with optimal participation in the intervention.
Ethics oversight	The CHU de Québec-Université Laval ethical review committee approved the research protocol. Written informed consent was obtained from all participants.

Note that full information on the approval of the study protocol must also be provided in the manuscript.

Field-specific reporting

Please select the one below that is the best fit for your research. If you are not sure, read the appropriate sections before making your selection.

☒ Life sciences ☐ Behavioural & social sciences ☐ Ecological, evolutionary & environmental sciences

For a reference copy of the document with all sections, see nature.com/documents/nr-reporting-summary-flat.pdf

Life sciences study design

All studies must disclose on these points even when the disclosure is negative.

Sample size	The sample size for the intervention was determined to detect a 20% reduction in plasma LDL-C levels (primary outcome) following the WFPB diet compared with the SAD, with analyses conducted separately by sex. Although we did not anticipate effect modification by sex, the trial was designed to ensure adequate power to detect clinically meaningful reductions in both males and females, thereby maximizing the potential for clinical translation, irrespective of sex. This expected effect size was a conservative estimate based on results from previous RCTs testing the impact of plant-rich diets on plasma lipids in individuals with non-familial hypercholesterolemia. We assumed that the standard deviation of the within-subject difference in LDL-C levels between diets would be similar to the mean treatment effect. Power calculations (GPower, v3.1.9.7) indicated that a sample size of 25 participants per sex would provide 80% power to detect such a difference at a two-sided alpha level of 5%. Accordingly, we aimed to have 25 female and 25 male participants complete the trial. We originally accounted for an anticipated 20% dropout rate in our recruitment strategy, based on previous trials by our group, meaning that up to 62 participants could be randomized, to ensure having 50 completing the trial.
Data exclusions	We conducted both intention-to-treat and per protocol analyses. In the per protocol analyses, two participants were excluded because they began medications known to affect lipid metabolism (isotretinoin and methylphenidate) during the study. Moreover, one participant dropped

out during the first week of the trial and could therefore not be included in the analytical models.

Replication	We did not replicate the findings. However, this study was a cross-over design, hence, every participants was used as its own control, which enhance the robustness of the findings.
Randomization	Randomization was done by research coordinators and was stratified by sex (female, male) and LDLR genotype (RN vs others), using 8 blocks of 8 subjects each, with an allocation ratio of 1:1.
Blinding	Participants and study coordinators could not be blinded to the interventions due to their nature. However, laboratory analyses were conducted in a blinded fashion, and investigators remained blinded to the intervention groups during statistical analyses until the final analyses of the primary outcome were completed.

Reporting for specific materials, systems and methods

We require information from authors about some types of materials, experimental systems and methods used in many studies. Here, indicate whether each material, system or method listed is relevant to your study. If you are not sure if a list item applies to your research, read the appropriate section before selecting a response.

Materials & experimental systems

n/a	Involved in the study	
☒	☐	Antibodies
☒	☐	Eukaryotic cell lines
☒	☐	Palaeontology and archaeology
☒	☐	Animals and other organisms
☐	☒	Clinical data
☒	☐	Dual use research of concern
☒	☐	Plants

Methods

n/a	Involved in the study	
☒	☐	ChIP-seq
☒	☐	Flow cytometry
☒	☐	MRI-based neuroimaging

Clinical data

Policy information about clinical studies

All manuscripts should comply with the ICMJE guidelines for publication of clinical research and a completed CONSORT checklist must be included with all submissions.

Clinical trial registration	This trial was registered at clinicaltrials.gov (NCT05181553) on December 20th, 2021.
Study protocol	The protocol is accesible on clinicaltrials.gov (NCT05181553)
Data collection	The study was conducted at the Institute of Nutrition and Functional Foods (INAF) of Laval University in Quebec City, Canada between January 2022 and June 2024. Volunteers were recruited through referral from physician collaborators at the Lipid Clinic of the CHU de Québec-Université Laval in Quebec City, Canada. To be eligible, participants had to be adults (18 to 60 years old) with genetically confirmed HeFH. Data on the FH-causing variant and LDLR genotype were obtained from medical records. Premenopausal female participants were required to have a regular menstrual cycle for more than 3 months and postmenopausal female participants were required to not use hormone replacement therapy. Exclusion criteria included any cardiovascular disease-risk exacerbating conditions (e.g., over 60 years old, homozygous FH, personal history of cardiovascular disease, diabetes/anti-diabetic drug use, severe obesity, unstable body weight for more than 3 months, uncontrolled hypertension and genetic hypertriglyceridemia), allergies or aversions to components of the experimental diets and any conditions that would interfere with optimal participation in the intervention.
Outcomes	The primary outcome is post-diet LDL-C levels and was measured using a Siemens Dimension Vista (Siemens Healthcare Limited, Germany)

Plants

Seed stocks	*Report on the source of all seed stocks or other plant material used. If applicable, state the seed stock centre and catalogue number. If plant specimens were collected from the field, describe the collection location, date and sampling procedures.*
Novel plant genotypes	*Describe the methods by which all novel plant genotypes were produced. This includes those generated by transgenic approaches, gene editing, chemical/radiation-based mutagenesis and hybridization. For transgenic lines, describe the transformation method, the number of independent lines analyzed and the generation upon which experiments were performed. For gene-edited lines, describe the editor used, the endogenous sequence targeted for editing, the targeting guide RNA sequence (if applicable) and how the editor was applied.*
Authentication	*Describe any authentication procedures for each seed stock used or novel genotype generated. Describe any experiments used to assess the effect of a mutation and, where applicable, how potential secondary effects (e.g. second site T-DNA insertions, mosiacism, off-target gene editing) were examined.*
