# Supplementary material for: Impact of a whole food, plant-based diet on LDL-cholesterol and cardiovascular risk factors in adults with heterozygous familial hypercholesterolemia: a randomized, two-period, two-treatment, crossover, fully controlled feeding trial
Source: Nat Commun. 2026 May 20;17:6632. doi: 10.1038/s41467-026-73468-4 (PMC13381664; doi:10.1038/s41467-026-73468-4)
Supplement: Supplementary file 3 — Transparent Peer Review file [file 41467_2026_73468_MOESM3_ESM.pdf]

nature portfolio

Peer Review File

Impact of a whole food, plant-based diet on LDL-cholesterol and cardiovascular risk factors in adults with heterozygous familial hypercholesterolemia: a randomized, two-period, two-treatment, crossover, fully controlled feeding trial

Corresponding Author: Dr Jean-Philippe Drouin-Chartier

This file contains all reviewer reports in order by version, followed by all author rebuttals in order by version.

Version 1:

Reviewer comments:

Reviewer #1

(Remarks to the Author)
This study addresses an important and overlooked aspect in the care of patients with familial hypercholesterolaemia. The main result is that a whole food, plant-based diet can effectively lower LDL-C and apoB in untreated adult patients with genetically 'untreated' FH.

The study design is at face value good, but on closer inspection the washout periods for previous drug use and active treatment during the trial are bordering on too short. The very short period of intervention does not simulate clinical practice and one cannot strictly employ an FH specific risk equation to predict what the long-term impact of the WFPB is because drug therapy would be added and the diet alone would not be sustained over 5 years.

The design would be more impactful if the study also included a parallel trial of patients on usual care or even a trial restricted to children and adolescents with FH (a group in whom drug therapy is especially overlooked) and their families. So, there is a concern about generalizability and relevance with this study design.

The use of genetically defined FH is good, and the attempt at a mechanistic explanation is laudable, noting the deficiencies of using sterol markers to reflect cholesterol absorption.

Certain sections of the manuscript are too long, like on pages 7, 10-16, 22-28.

The references are adequate, but there might have been earlier regression studies showing CAD benefit of diet in adults with phenotypic FH.

(Remarks on code availability)
No comment

Reviewer #2

(Remarks to the Author)
For authors:

Strengths include the novel question in this important population, careful feeding protocol, cross-over randomized design, and lack of phytosterol fortification.

However, several aspects of the manuscript are reducing its validity and value, standing in the way of its full positive impact. In particular, the authors place far too much emphasis on secondary and exploratory findings; highlight nonsignificant findings that should only be described as such; and include highly speculative discussion and statements not supported by

the findings.

There is no need to “oversell” the paper, which only weakens it. The design is strong, and the primary findings important. Careful revisions are needed throughout to focus on the main findings, reduce focus on secondary and exploratory findings (and all nonsignificant findings), and remove speculation.

Major comments:

Abstract:
The body weight change should be added; and that results were adjusted for change in BMI. The post-intervention biomarker analyses are highly exploratory; delete.

Introduction:
Lines 53-55: Predimed was not a trial low in red and processed meats and high in minimally processed plant foods. Red and processed meat consumption did not significantly change between the groups. The only major change was higher intake of plant fats from nuts and EVOO. Other plant foods changed only modestly, e.g. vegetables increased by only 8 g/d, fruits increased by only 6 g/d; no change in legumes or whole grains. This text requires significant revision.

Lines 57-59: These references are likewise presented incorrectly. Ref 14 does not show that a diets low in red and processed meats and high in minimally processed plant foods positively affects CVD risk factors. In fact, it shows minimal and inconsistent effects of red meat consumption on CVD risk factors. This citation is important and needs to be presented accurately.

Methods:
The exclusion of adults age 60+ is unjustified. These individuals should not have differing dietary responses, and also are at higher absolute risk and likely to benefit. Needs justification.

It’s unfortunate the WFPB diet was matched to total fat (it was not matched on many other relevant macronutrients). Predimed and many other studies demonstrate the CVD benefits of higher fat diets that are abundant in plant-derived unsaturated fats. The benefits of a higher fat WFPB would likely have been even greater; needs discussion.

In a cross-over design, why were models matched for age, sex, and LDLR? Individuals are compared to themselves, these factors are not varying.

Lines 330-344: The “determinant” analyses should be renamed. If this is aiming to evaluate effect modifiers, then baseline variables are reasonable. However, baseline variables cannot be determinants. If aiming to understand mediators, all of the time-varying biomarkers could simply be downstream correlates, so none of these can be confirmed as determinants (mediators). They are correlates.

Many different outcomes and analyses are listed (lines 279-368). The text should clearly specify which of these were prespecified primary, secondary, or exploratory outcomes; and which were post-hoc. (some are noted as a priori; many others are not specified)

The original sample size calculation aimed to enroll 62 individuals to account for drop-out; but only 50 were enrolled. Why was this changed?

There is very limited evidence that methylphenidate affects LDL-C; this participant should likely not be excluded from the per protocol analysis.

Results:
Line 440: Change to “Correlates of…” Lines 441-455: Delete any mention of “potential association” or ‘tended to be” findings. If $P<0.05$, there is no significant association, period. (esp. for these secondary analyses with multiple comparisons, which should really be corrected for false discovery) There should be no “reduced model” – the primary model should be used only.

Single person responses can be due to change; this is why we do trials with many people combined. Move Fig 2 to the supplement.

The lack of effect on CRP on glucose-insulin control is striking. This requires significant discussion and consideration, given differences with prior studies. Perhaps duration was too brief.

Effect modification analyses were prespecified. Table S6 should be moved to the main tables.

Lines 425-428: P=0.13 (and not even corrected for multiple secondary comparisons). Edit text to “was not significantly different”, and delete Fig S2.

Line 438-439: no significant effect on either GLP-1 or PYY; edit text accordingly (delete ‘tended to increased”). P=0.09, and not corrected for multiple secondary comparisons.

Table 5: move to supplement. Suggest correct all the findings here for multiple comparisons to avoid false discovery.

Discussion:
Lines 491-493: move out of primary summary paragraph, which should focus on the main findings. These can be mentioned very briefly later in the discussion as highly exploratory preliminary findings, with unclear relevance that should be considered hypothesis generating and require confirmation. Line 495: delete from summary paragraph, this is speculative.

Lines 512-520: Appropriate text, well done. Add that future studies may wish to test WFPB diets that have more non-meat sources of saturated fat, such as dairy fat, cocoa, or coconut fat that are less abundant in 16:0, which may have similar benefits on other risk markers without lowering HDL-C and ApoA1.

Line 522; add “predicted” risk. Line 529: delete or edit, a clinically meaningful effect on CVD risk was not shown.

Very low SFA diets have been consistently associated with risk of stroke, potentially linked to increased cerebral vascular fragility. This should be added and discussed as a potential long-term risk.

Lines 530-534: This is the crux of the limitations. Needs more discussion; for example, could similar findings have been seen on meds?

Lines 535-542: Delete. You had absolutely no power, nor any long term follow-up, to understand the potential for underdiagnosis. A difference of 1 or 2 people out of 10 has zero implications for diagnostic thresholds.

Lines 543-546: delete, simply repeating the results; and these findings could very likely be chance.

Line 551-552: delete. absence of significant effect modification (failure to reject null) is not the same as confirmation of beneficial effects (confirming the alterative hypothesis).

Lines 558-658: delete. See earlier comments.

Lines 577-588: delete, highly speculative.

Line 589: delete “important”, there is no evidence this was “important.” Delete repetition of results text, e.g. lines 590-591, throughout the discussion. Line 591-593: speculative, soften language. Line 593-594: change to “However, the lack of significant effect on GLP-1 does not support this effect on appetite.” Lines 594-599 delete, highly speculative.

Line 600: change “clinically meaningful” to “possible”. Delete repeated results text, which s most of the rest of the paragraph. Line 625-626: only a limitation, not a strength; edit.

Line 634: suggest delete comment on inter-individual variability (you have not shown this is due to anything other than chance, which is a normal part of the world).

Line 620: delete.

Minor comments:

Lines 134-143: “strictly forbidden”, “not allowed” , “only allowed” , “had to”, “not permitted” all need editing. These were free-living participants, they may have been advised or instructed but cannot have been forbidden, not allowed, or forced.

Lines 218-263: these were secondary or exploratory outcomes; move text to supplementary materials.

Results, first paragraph: The % who stopped prior meds (and which meds) should be specified in the text.

(Remarks on code availability)
n/a

Reviewer #3

(Remarks to the Author)
The objectives, rationale and study design are sound. The study has been well conducted, and appropriate statistics methods have been applied and competently executed in a transparent manner. The trialists have done well to achieve compliance and encourage patients to complete the study with very few missing observations or patient dropouts.

The paper is worthy of publication with only minor edits which I outline below.

In the title and elsewhere state the number of periods and treatments used in the crossover design, either in full as “two-period, two-treatment crossover”, or as an “AB/BA crossover” design.

On line 24 of the abstract under methods please quote the number of patients assigned to each treatment sequence, e.g., see highlighted addition below:
“ .. confirmed HeFH, free of cholesterol-lowering medication, consumed a WFPB and a SAD for 4 weeks each in a random order, (27 to receive SAD first and 23 WFPB first), under fully controlled, isocaloric feeding conditions."

The abstract should also identify the primary as opposed to secondary outcome/objective. This can be done succinctly, by modifying line 21 under the heading Objective to
“(SAD), on the primary outcome of LDL-cholesterol (LDL-C), and other CVD risk factors”.

The abstract should state or reference the source of funding.

In the main text under the section “Study design” add a sentence to explain the rationale for using a cross-over design, e.g., something along the lines of the following, perhaps with a scientific reference.
“A cross-over design is more efficient way of measuring a treatment effect as each patient acts as their own control and a smaller sample size is required. The study laboratory parameters are expected to be affected relatively quickly by changes in diet, and a 2-to-4-week wash-out period is expected to more than adequate to avoid a carry-over effect.”

The choice of a linear mixed model with a random intercept for each patient is a good one, as it allows for correlations between observations on the same patient with each acting as their own control; the choice of the restricted maximum likelihood to fit these models is also optimal.
In fact, the methods section should include a more precise description of the models, e.g.,
“Linear mixed models were fitted to the primary and secondary outcomes using restricted maximum likelihood with a random intercept term for each patient to allow for correlation between observations on the same patient so that each patient acts is its own control.”

It is worth stating that the linear mixed models allow for unbalanced data and for patients missing a data point to be retained in the analysis.
Although page 15 mentions a period*diet interaction, please make it clear in the methods that an apriori decision was made to adjust for the period effect so that this is clear at a glance.

Please include a table, at least in the supplement, that summarises the data with two rows for the two treatment sequences AB , BA and columns from right to left for the number of patients at the start n, the number of observations (patients with measurements), Mean (SD) LDL-c reading at each time point, at the start and end of each period.

Two minor points.
Please provide a more detailed sentence on how the stratified randomisation was carried out.
The study sample size appears correct, but for completeness please specify the precise input/options used in the G*power sample size calculation.

(Remarks on code availability)
The code is logical, accurate and well annotated so that the analysis can easily be reproduced.

Version 2:

Reviewer comments:

Reviewer #1

(Remarks to the Author)
The authors have responded adequately to concerns raised

(Remarks on code availability)

Reviewer #2

(Remarks to the Author)
For authors:

The authors partly responded to some of the concerns, but unfortunately not to other major comments. For example, they continue to equivocally present null findings and ignore potential risks in their discussion. Attention to these issues is needed.

Major comments:

Introduction: As noted previously, some references are described incorrectly. Ref 14 does not show that a diets low in red and processed meats and high in minimally processed plant foods positively affects CVD risk factors. In fact, it shows minimal and inconsistent effects of red meat consumption on CVD risk factors. The latter finding needs to be specified (not ignored or deleted) and presented accurately.

Methods:
As noted previously, the exclusion of adults age 60+ needs justification. The authors provided an explanation in the response letter but not in the actual manuscript. The justification should be added: "The age threshold was driven by the requirement to discontinue cholesterol-lowering medication. Given the higher baseline CVD risk in older adults, we excluded participants aged ≥60 years as a risk-mitigation measure, based on safety considerations related to temporary treatment withdrawal."

As noted previously, many different outcomes and analyses are listed (lines 279-368). The text should clearly specify, in once place, which of these were prespecified primary, secondary, or exploratory outcomes; and which were post-hoc. (some are noted as a priori; many others are not specified). The authors point to supplementary text, but it does not contain this information. The text should clearly specify, in the main paper, which of the outcomes were prespecified primary, secondary, or exploratory outcomes; and which were post-hoc

Results:
As noted previously, the authors should be clear about results that were not statistically significant. Lines 141-143 should read, "The proportion of participants whose LDL-C levels exceeded the thresholds warranting investigation… was not significantly different on the WFPB diet versus the SAD diet (79.2% versus 87.5, P=0.13). As before, I strongly recommend deleting the associated supplementary figure, which is not needed and unnecessarily emphasizes a nonsignificant findings

Similarly, for the effects on GLP-1 or PYY, the text should specify that no significant effects were seen (rather than just reporting the differences)

Line 160: As noted previously, a single correlation with P=0.04 among many exploratory findings is unlikely to be meaningful and more likely a chance finding. Using declarative text "was associated with" is not appropriate here, without further caveats. Add here: "… P = 0.04), although this exploratory finding was not adjusted for multiple comparisons and may have been due to chance."

As noted previously, very low SFA diets have been consistently associated with risk of stroke, potentially linked to increased cerebral vascular fragility. This has been seen irrespective of the replacement nutrient. The diet used here is in the range where higher risk was identified. This literature and potential risk cannot be ignored. This should be added to the discussion as a potential long-term risk.

Abstract: My comment to add the body weight change is not based on either its presence or lack of clinical or statistical significance. This is a relevant outcome to interpret all the findings, and should be included in the abstract.

(Remarks on code availability)

Reviewer #3

(Remarks to the Author)
As I reported earlier when reviewing the first draft, the analysis methods are appropriate and correctly executed; the study results noteworthy and add to research in the related field.
By responding to valuable, detailed comments from the other reviewers, including a suggestion to place less emphasis on secondary endpoints and make more qualified statements when interpreting the results,
the authors now present a much improved version of the paper.

I am satisfied with the authors' response to my comments and, provided the other reviewers are happy with each of the individual responses to their comments, I suggest that the paper is ready for publication.

(Remarks on code availability)
The code is written very clearly and the comments in the code are enough to understand what exactly has been done without the need to even look at a readme. I did not actually re-run the code as that would require access to the individual level data and would not be appropriate. However, the authors use standard R packages and write their code in a very transparent way which would make it easy to reproduce the results.

Version 3:

Reviewer comments:

Reviewer #2

(Remarks to the Author)

(Remarks on code availability)

Open Access This Peer Review File is licensed under a Creative Commons Attribution 4.0 International License, which permits use, sharing, adaptation, distribution and reproduction in any medium or format, as long as you give appropriate credit to the original author(s) and the source, provide a link to the Creative Commons license, and indicate if changes were made.
In cases where reviewers are anonymous, credit should be given to 'Anonymous Referee' and the source.
The images or other third party material in this Peer Review File are included in the article's Creative Commons license, unless indicated otherwise in a credit line to the material. If material is not included in the article's Creative Commons license and your intended use is not permitted by statutory regulation or exceeds the permitted use, you will need to obtain permission directly from the copyright holder.
To view a copy of this license, visit https://creativecommons.org/licenses/by/4.0/

We would like to thank the editorial team of *Nature Communications* and the reviewers for their constructive comments. We have been able to address all the reviewers' concerns and make the appropriate changes in the revised manuscript. Please find below our point-by-point response to comments. Please note that, for clarity, some comments were divided into multiple parts to facilitate our responses. We did not modify the wording or meaning of the comments in the process. Also, we reordered the manuscript sections in accordance with the journal requirements (i.e., Results and Discussion before the Methods), and corrected some typos we detected reviewing the paper. All authors agree with the changes made to the paper.

Reviewer 1

This study addresses an important and overlooked aspect in the care of patients with familial hypercholesterolemia. The main result is that a whole food, plant-based diet can effectively lower LDL-C and apoB in untreated adult patients with genetically 'untreated' FH.

Response: We thank the reviewer for recognizing the relevance of our study and taking the time to review and comment our work.

The study design is at face value good, but on closer inspection the washout periods for previous drug use and active treatment during the trial are bordering on too short.

Response: Participants were asked to discontinue all cholesterol-lowering medications four weeks before the start of the feeding intervention and to remain off therapy throughout the trial. Pharmacokinetic and pharmacodynamic studies of statins, ezetimibe, and PCSK9 monoclonal antibodies (none of the participants was receiving inclisiran at the time of the study) indicate half-lives ranging from less than 1 day to 17 days, with LDL-C levels generally returning to untreated levels within 28 days after the last dose.

Please see:
1. Lennernäs H, Fager G. Pharmacodynamics and pharmacokinetics of the HMG-CoA reductase inhibitors. Similarities and differences. Clinical pharmacokinetics 1997;32:403-25.
2. Simard C, Turgeon J. The pharmacokinetics of ezetimibe. The Canadian journal of clinical pharmacology 2003;10 Suppl A:13a-20a.
3. Kasichayanula S, Grover A, Emery MG, et al. Clinical Pharmacokinetics and Pharmacodynamics of Evolocumab, a PCSK9 Inhibitor. Clinical pharmacokinetics 2018;57:769-79.

We clarified these elements in the methods of the revised paper.

Please see line 338: *"A 4-week washout period of cholesterol-lowering medication is sufficient for LDL-C levels to return to untreated values.[49-51]"*

Moreover, our analyses focused on outcome levels measured at the end of each fully controlled feeding period. Thus, at the time of sampling, cholesterol-lowering medications had been discontinued for at least 8 weeks, which is more than sufficient to eliminate any residual effect. In addition, because diet sequence was randomized, any residual carryover effect of these medications would have been expected to appear as a sequence effect: the diet effect would have been smaller in the SAD–WFPB sequence and larger in the WFPB–SAD sequence. However, no sequence effect was observed. Please see **Supplementary Table 7**.

Finally, we interpreted the reviewer's comment on "active treatment" as referring to the dietary intervention. Four-week periods of fully controlled feeding are well established to induce clinically significant changes in plasma LDL-C and other outcomes of interest, as demonstrated in numerous prior studies.

Please see:

1. Gardner CD et al., The effect of a plant-based diet on plasma lipids in hypercholesterolemic adults: a randomized trial. Ann Intern Med. 2005 May 3;142(9):725-33.
2. Jenkins DJA et al., A dietary portfolio approach to cholesterol reduction: combined effects of plant sterols, vegetable proteins, and viscous fibers in hypercholesterolemia. Metabolism. 2002 Dec;51(12):1596-604.
3. Jenkins DJA et al., Direct comparison of a dietary portfolio of cholesterol-lowering foods with a statin in hypercholesterolemic participants. Am J Clin Nutr. 2005 Feb;81(2):380-7.

The very short period of intervention does not simulate clinical practice.

Response: We agree with the reviewer. This study was designed as an *efficacy* trial, intended to test whether the diet has an impact under strictly controlled conditions, rather than as an *effectiveness* trial aimed at simulating routine clinical practice. We have clarified this positioning when stating the study objective at the end of the Introduction.

Please see line 72: *"This randomized, two-period, two-treatment, crossover, fully controlled feeding, efficacy trial aimed to evaluate the impact of a whole food plant-based diet (WFPB), compared with a standard American diet (SAD), on plasma LDL-C levels (primary outcome), other cardiovascular risk factors and estimated 10-year CVD risk in adults with genetically confirmed HeFH."*

One cannot strictly employ an FH specific risk equation to predict what the long-term impact of the WFPB is because drug therapy would be added and the diet alone would not be sustained over 5 years.

Response: We agree with the reviewer that it would be an overinterpretation to extrapolate our findings to long-term, real-world effects of a WFPB diet. We interpret this comment as closely related to the prior on the duration of the interventions and take this opportunity to reiterate that this study was designed as an efficacy trial (effects under strictly controlled conditions), not an effectiveness trial intended to simulate clinical practice.

We consider it valuable to report the effect of the WFPB diet on the FH Risk Score, which provides an estimate of the 10-year risk of CVD in adults with HeFH. Reporting this score helps contextualize the dietary effect beyond LDL-C alone, because the algorithm incorporates multiple risk factors (https://doi.org/10.1161/ATVBAHA.121.316106). Importantly, this score was specifically developed and validated for adults with FH, addressing repeatedly noted limitations of general-population risk scores (e.g., Framingham) in this population. In addition, the FH Risk Score leverages LDL-C values obtained without lipid-lowering therapy, which aligns well with our trial design.

Reviewer #2 raised a similar comment (please see below). We therefore carefully revised the entire manuscript to avoid overinterpreting the diet-induced changes in the FH Risk Score.

The design would be more impactful if the study also included a parallel trial of patients on usual care or even a trial restricted to children and adolescents with FH (a group in whom drug therapy is especially

overlooked) and their families. So, there is a concern about generalizability and relevance with this study design.

Response: We agree with the reviewer that such trials would be a valuable addition to the field. However, the objective of our study was to test the effect of a WFPB diet on LDL-C and other CVD risk factors in adults with HeFH under strictly controlled conditions (i.e., an efficacy trial), to address a key knowledge gap: whether diet has a clinically meaningful effect in this population. We believe our findings are robust and clinically relevant and warrant publication as a dedicated report. Within the scope of the current revision, we were not able to add parallel trials as suggested. Nonetheless, we have added a statement in the Discussion to highlight the proposed investigations as important directions for future research.

Please see line 281: *"Additional trials are also warranted to determine whether a WFPB diet has clinically meaningful effects on CVD risk factors in children and adolescents with HeFH, as well as in older adults aged >60 years, which were not included in the present trial."*

The use of genetically defined FH is good, and the attempt at a mechanistic explanation is laudable, noting the deficiencies of using sterol markers to reflect cholesterol absorption.

Response: Thank you.

Certain sections of the manuscript are too long, like on pages 7, 10-16, 22-28.

Response: Regarding page 7 of the original manuscript, which describes the composition of the experimental diets, we believe this section is central to our work and essential for enabling replication of our results by other research teams. We therefore kept it in the main manuscript. However, we moved all methodology related to appetite hormones, PCSK9, noncholesterol sterols, subjective appetite sensations, and diet appreciation to the Supplementary Material (originally pages 10–16). We also shortened the Discussion from nearly 7 pages to 5 pages.

The references are adequate, but there might have been earlier regression studies showing CAD benefit of diet in adults with phenotypic FH.

Response: We agree that earlier studies have reported cardiovascular benefits of dietary interventions in adults with phenotypic FH. A key feature of our study focused is the inclusion of individuals with *genetically* confirmed HeFH. In line with the reviewer's comment, we compared our findings in the Discussion with those from a meta-analysis of RCTs evaluating the Portfolio diet in individuals with hyperlipidemia, including phenotypic FH.

Please see line 199: *"The 18% decrease in LDL-C and 15% reduction in ApoB observed closely mirror the findings of a meta-analysis of dietary intervention studies on the Portfolio diet–specifically designed to lower LDL-C[24]–which reported reductions of 17% and 15% in LDL-C and ApoB across seven RCTs in individual with hyperlipidemia, including phenotypic, but not genetically confirmed HeFH.[25]"*

Reviewer 2

Strengths include the novel question in this important population, careful feeding protocol, cross-over randomized design, and lack of phytosterol fortification.

However, several aspects of the manuscript are reducing its validity and value, standing in the way of its full positive impact. In particular, the authors place far too much emphasis on secondary and exploratory findings; highlight nonsignificant findings that should only be described as such; and include highly speculative discussion and statements not supported by the findings.

There is no need to “oversell” the paper, which only weakens it. The design is strong, and the primary findings important. Careful revisions are needed throughout to focus on the main findings, reduce focus on secondary and exploratory findings (and all nonsignificant findings), and remove speculation.

Response: We thank Reviewer 2 for the time and interest devoted to our manuscript, as well as for their recommendations to improve the quality of the article. We agree that excessive emphasis was placed on some exploratory findings, and we revised the manuscript accordingly in response to their comments.

Major comments:

Abstract:
The body weight change should be added; and that results were adjusted for change in BMI. The post-intervention biomarker analyses are highly exploratory; delete.

Response: We deleted the post-intervention biomarker results from the Abstract as requested. Regarding within-diet body weight change, we appreciate the reviewer’s suggestion; however, we only added a mention that the analyses were adjusted for it; we did not add the result *per se*. The reason is that even though the between-diet difference, although statistically significant, it was small and unlikely to be clinically meaningful (–0.48 %; 95% CI: –0.92 to –0.05; *P*=0.03). Indeed, in the model on the effect of diet on LDL-C (primary outcome), difference in within-diet body weight change had no statistically significant effect as a covariable (*P*=0.97). We report weight change in the Results section and adjusted our models to account for potential confounding. We believe this approach keeps the Abstract focused on the primary clinically relevant outcome, while ensuring transparency in the abstract and the full manuscript.

Please see the abstract, line 27: *“In fully adjusted models controlling for BMI at the end of each diet, weight change during each intervention, and diet sequence, the WFPB induced a clinically significant reduction in LDL-C relative to the SAD (−17.9%, 95% CI: −21.7% to −14.3%; P<0.0001).”*

Introduction:

Lines 53-55: Predimed was not a trial low in red and processed meats and high in minimally processed plant foods. Red and processed meat consumption did not significantly change between the groups. The only major change was higher intake of plant fats from nuts and EVOO. Other plant foods changed only modestly, e.g. vegetables increased by only 8 g/d, fruits increased by only 6 g/d; no change in legumes or whole grains. This text requires significant revision.

Response: We thank the reviewer for this important correction. Accordingly, we deleted the sentence and reference to PREDIMED to avoid any misinterpretation.

Lines 57-59: These references are likewise presented incorrectly. Ref 14 does not show that a diet low in red and processed meats and high in minimally processed plant foods positively affects CVD risk factors. In fact, it shows minimal and inconsistent effects of red meat consumption on CVD risk factors. This citation is important and needs to be presented accurately.

Response: We thank the referee for the remark. We modified the text and the references to accurately support the claim.

Please see line 55: *"In the shorter term, randomized controlled trials (RCTs) have shown that such dietary patterns, or some of their components, such as replacing red meat with high-quality plant protein sources (e.g., legumes, soy, and nuts), positively affect CVD risk factors, including atherogenic plasma lipids.[13-16]"*

Methods:
The exclusion of adults age 60+ is unjustified. These individuals should not have differing dietary responses, and also are at higher absolute risk and likely to benefit. Needs justification.

Response: We agree with the reviewer that adults aged ≥60 years have a higher absolute cardiovascular risk and may therefore derive substantial absolute benefit from dietary interventions. The age threshold was not chosen because we anticipated a different biological response to the diet by age. Rather, it was driven by the requirement to discontinue cholesterol-lowering medication. Given the higher baseline CVD risk in older adults, we excluded participants aged ≥60 years as a risk-mitigation measure, based on safety considerations related to temporary treatment withdrawal.

As the text already presents age ≥60 years as a condition associated with increased CVD risk when listing inclusion/exclusion criteria, we did not make changes to this section to avoid repetition.

Please see line 325: *"Exclusion criteria included any CVD-risk exacerbating conditions (e.g., over 60 years old, homozygous FH, personal history of cardiovascular disease, diabetes/anti-diabetic drug use, severe obesity, unstable body weight for more than 3 months, uncontrolled hypertension and genetic hypertriglyceridemia), allergies or aversions to components of the experimental diets and any conditions that would interfere with optimal participation in the intervention."*

However, we added a mention, in the discussion, on the need to confirm the benefits observed in the present study in older adults aged >60 years,

Please see line 281: *"Additional trials are also warranted to determine whether a WFPB diet has clinically meaningful effects on CVD risk factors in children and adolescents with HeFH, as well as in older adults aged >60 years, which were not included in the present trial."*

It's unfortunate the WFPB diet was matched to total fat (it was not matched on many other relevant macronutrients). Predimed and many other studies demonstrate the CVD benefits of higher fat diets that are abundant in plant-derived unsaturated fats. The benefits of a higher fat WFPB would likely have been even greater; needs discussion.

Response: We thank the reviewer for this comment. Designing experimental diets inevitably requires a series of decisions and compromises. In this trial, we chose to make the two diets isoenergetic and to match them for the three macronutrients, fat (35% of energy), protein (15%), and carbohydrate (50%). On the one hand, observing a mean 17.9% reduction in LDL-C despite this macronutrient matching shows that a WFPB diet can confer clinically meaningful benefits even when total macronutrient distribution is held constant relative to the control diet. On the other hand, this design choice raises an important question, as mentioned by the reviewer: whether this reflects the maximal achievable diet-induced benefit, or whether a different macronutrient distribution could have produced larger effects.

We agree that PREDIMED demonstrated cardiovascular benefits of dietary patterns rich in plant-derived unsaturated fats. In PREDIMED, participants assigned to the Mediterranean diet supplemented with extra-virgin olive oil reported a mean of 28.2% of energy from unsaturated fat (22.1% MUFA and 6.1% PUFA), while those assigned to the Mediterranean diet supplemented with nuts reported 28.6% (20.9% MUFA and 7.7% PUFA). These values are broadly comparable to the fatty acid profile of our WFPB diet, which provided 26% of energy from unsaturated fat (16.5% MUFA and 9.5% PUFA).

A key difference between PREDIMED and our WFPB intervention relates to the carbohydrate contribution and associated fiber intake. In PREDIMED, carbohydrate intake was approximately 40% of energy, whereas our WFPB diet provided 50%. Consistent with this, fiber intake was approximately 25–27 g/day in PREDIMED, while our WFPB diet provided 47.5 g/2500 kcal, with a soluble-to-insoluble fiber ratio >2:1. Because soluble fiber is known to lower LDL-C, part of the observed effect may plausibly be attributable to the high soluble fiber content of the WFPB diet.

Overall, we agree that a higher intake of plant-derived unsaturated fat could plausibly yield additional LDL-C lowering. We have now added this point to the revised Discussion.

Please see line 219: *"Likewise, future studies could examine whether a WFPB diet with a different macronutrient distribution, such as a higher content of plant-derived unsaturated fatty acids similar to that tested in the PREDIMED trial[30], would confer greater benefits on plasma lipids and other CVD risk factors than those observed in the present study."*

In a cross-over design, why were models matched for age, sex, and LDLR? Individuals are compared to themselves, these factors are not varying.

Response: We thank the reviewer for pointing out this issue. We agree that, in such models, these covariables are not informative because the factors do not vary within-individuals. We therefore removed the adjustment for age, sex, and LDLR genotype from our analyses. This change had virtually no impact on the results or their interpretation. However, some estimates slightly changed, this explains the changes seen in the revised tables.

Lines 330-344: The "determinant" analyses should be renamed. If this is aiming to evaluate effect modifiers, then baseline variables are reasonable. However, baseline variables cannot be determinants. If aiming to understand mediators, all of the time-varying biomarkers could simply be downstream correlates, so none of these can be confirmed as determinants (mediators). They are correlates.

Response: Thank you for this comment. We renamed the analyses as "correlate analyses", as suggested.

Please see line 519 (methods): *"Third, we explored the correlates of the LDL-C response to the WFPB (post hoc analysis). Using the stats package (v4.3.0), we fitted a simple linear regression model including as potential correlates: unmodifiable factors (sex, age, LDLR genotype), lifestyle-related factors (BMI, as a surrogate of energy balance[47], and mean calorie intake), cardiometabolic health surrogates (CRP, HOMA-IR, fasting total GLP-1, systolic blood pressure), and cholesterol and lipoprotein-metabolism related factors (lathosterol/cholesterol ratio, campesterol/cholesterol ratio, and levels of PCSK9, Lp(a) and LDL-C). For all potential correlates, we used values obtained at the end of the SAD phase to simulate the metabolic state of individuals before adopting a WFPB. Partial R^2 values were calculated using the rsq package (v2.6). The LDL-C response was defined as the percent difference between post-WFPB and*

post-SAD levels and was treated as the dependent variable. Each potential correlate was treated as the independent variable. These analyses were repeated on ApoB response.“

Please see line 157 (results): *“Correlates of dietary response. No evidence of association between any potential correlates and LDL-C response to the WFPB was found (**Supplementary Table 7**). For ApoB response, each 1 pmol/L-increment in fasting total GLP-1 was associated with a –0.15% decrease in ApoB diet-induced changes (95% CI = –0.31% to –0.01%, P = 0.04). The partial R^2 associated with fasting total GLP-1 was 12.6%.”*

Many different outcomes and analyses are listed (lines 279-368). The text should clearly specify which of these were prespecified primary, secondary, or exploratory outcomes; and which were post-hoc. (some are noted as a priori; many others are not specified)

Response: We added clarification to clearly specify whether each outcome were primary or exploratory and pre-specified or *post hoc*.

Please see the revised statistical analysis section starting line 463.

The original sample size calculation aimed to enroll 62 individuals to account for drop-out; but only 50 were enrolled. Why was this changed?

Response: Our sample size calculation indicated that 50 completers were required. We therefore set a recruitment target of 62 participants to allow for an anticipated 20% dropout, based on our experience with previous trials of similar design. However, actual drop-out rate was much lower. We were able to achieve 50 completers by recruiting only 51 participants. Accordingly, the study retained the planned statistical power despite enrolling fewer participants than the conservative recruitment target. We clarified this in the text.

Please see line 460: *“We originally accounted for an anticipated 20% dropout rate in our recruitment strategy, based on previous trials by our group.*[67] *As such, up to 62 participants (31 per sex) could be randomized to achieve 50 trial completers.”*

There is very limited evidence that methylphenidate affects LDL-C; this participant should likely not be excluded from the per protocol analysis.

Response: We agree with the reviewer that the evidence is limited. However, we are not comfortable retaining this participant in the per protocol analysis, as suggested. Available evidence suggests a favorable effect of this medication on LDL-C, and this participant experienced a decrease in LDL-C, raising concern that the outcome could have been influenced by methyphenidate rather than diet alone.

For full transparency to readers, the editorial board, and reviewers, we therefore applied our predefined criteria: initiation of any medication during the intervention period was considered a protocol deviation, and the participant was excluded accordingly.

Results:
Line 440: Change to “Correlates of…” Lines 441-455: Delete any mention of “potential association” or ‘tended to be” findings. If P<0.05, there is no significant association, period. (esp. for these secondary analyses with multiple comparisons, which should really be corrected for false discovery) There should be no “reduced model” – the primary model should be used only.

Response: We revised this section and the associated table to address the reviewer's concerns. First, we changed the wording from "determinants" to "correlates" throughout. Second, we removed the data-driven reduced-model selection to avoid over-interpretation and updated the Methods accordingly. We now present solely the full model for LDL-C and ApoB response.

In the Results, we rewrote the text to remove language such as "potential association" and "tended to," and we now only report the significant association between ApoB response and post-SAD GLP-1. Given the exploratory nature of these analyses, we also moved the table to the Supplementary material (**Supplementary Table 7**).

Please see line 157: *"Correlates of dietary response. No evidence of association between any potential correlates and LDL-C response to the WFPB was found (**Supplementary Table 7**). For ApoB response, each 1 pmol/L-increment in fasting total GLP-1 was associated with a –0.15% decrease in ApoB diet-induced changes (95% CI = –0.31% to –0.01%, P = 0.04). The partial R^2 associated with fasting total GLP-1 was 12.6%."*

We did not apply a formal adjustment for multiple comparisons in line with the recommendations of Schulz and Grimes on multiplicity in randomized trials (DOI: 10.1016/S0140-6736(05)66461-6). The text transparently reports the effect size to allow readers to interpret this result appropriately.

Single person responses can be due to change; this is why we do trials with many people combined. Move Fig 2 to the supplement.

Response: Done. It is now **Supplementary Figure 1**.

The lack of effect on CRP on glucose-insulin control is striking. This requires significant discussion and consideration, given differences with prior studies. Perhaps duration was too brief.

Response: We agree that the lack of effect on CRP and glycemic/insulin markers warrants discussion in light of prior studies. We expanded the Discussion to note that our cohort had largely normal baseline CRP levels and glucose homeostasis, which likely limited the potential for improvement. In addition, the relatively short intervention duration and the absence of substantial weight loss may have reduced the likelihood of detecting changes in inflammation and glucose homeostasis compared with studies enrolling individuals with insulin resistance and/or elevated inflammation, or using longer interventions. These points are now discussed in the limitations section.

Please see line 288: *"Third, most participants had normal CRP and glucose homeostasis, limiting the potential for improvement with WFPB diet. In addition, changes in these parameters are more consistently observed in cohorts with elevated baseline values and/or with longer interventions and/or meaningful weight loss, which may partly explain why we observed not effect."*

Effect modification analyses were prespecified. Table S6 should be moved to the main tables.

Response: Done. It is now **Table 3**.

Lines 425-428: P=0.13 (and not even corrected for multiple secondary comparisons). Edit text to "was not significantly different", and delete Fig S2.

Response: We edited the text. However, although this analysis is exploratory and should be interpreted with caution, we chose to retain it in the Supplementary Material (now **Supplemental Figure 2**).

Please see line 141: *"The proportion of participants whose LDL-C levels exceeded the thresholds warranting investigation for FH according to the Simplified Canadian Definition for FH[23] (≥4.50 mmol/L for 18 to 40 years, and >5.00 mmol/L for over 40 years) was 79.2% after the WFPB diet and 87.5% after the SAD diet (P=0.13;* ***Supplementary Figure 2****)."*

Line 438-439: no significant effect on either GLP-1 or PYY; edit text accordingly (delete 'tended to increased"). P=0.09, and not corrected for multiple secondary comparisons.

Response: We edited the text.

Please see line 153: *"Regarding appetite hormones, mean changes induced by the WFPB relative to the SAD were 3.4% (95% CI = –0.6% to 7.4%; P = 0.09) for fasting total GLP-1 and –2.6% (95% CI = –7.0% to 1.7%; P = 0.23) for peptide YY."*

Table 5: move to supplement. Suggest correct all the findings here for multiple comparisons to avoid false discovery.

Response: We moved the table to the Supplementary Material, where it is now presented as **Supplemental Table 7**. In the revised manuscript, we labelled this analysis as exploratory and hypothesis-generating. Accordingly, and in line with the recommendations of Schulz and Grimes on multiplicity in randomized trials (DOI: 10.1016/S0140-6736(05)66461-6) , we did not apply a formal adjustment for multiple comparisons. The text transparently reports the effect size to allow readers to interpret this result appropriately.

Discussion:
Lines 491-493: move out of primary summary paragraph, which should focus on the main findings. These can be mentioned very briefly later in the discussion as highly exploratory preliminary findings, with unclear relevance that should be considered hypothesis generating and require confirmation. Line 495: delete from summary paragraph, this is speculative.

Response: Done.

Lines 512-520: Appropriate text, well done. Add that future studies may wish to test WFPB diets that have more non-meat sources of saturated fat, such as dairy fat, cocoa, or coconut fat that are less abundant in 16:0, which may have similar benefits on other risk markers without lowering HDL-C and ApoA1.

Response: Thank you. The sentence is added.

Please see line 216: *"Future studies could test WFPB dietary patterns with alternative fat profiles, such as greater contributions from non-animal sources of saturated fat (e.g., cocoa, and coconut fat), to determine whether LDL-C and ApoB benefits can be maintained while attenuating decreases in HDL-C and ApoA1."*

Line 522; add "predicted" risk.

Response: We added "estimated", which we consider better reflects the type of information the FH risk Score provides.

Please see line 225: *"Specifically, relative to the SAD, the WFPB reduced by 11.9% the estimated 10-year CVD risk."*

Line 529: delete or edit, a clinically meaningful effect on CVD risk was not shown.

Response: Done.

Very low SFA diets have been consistently associated with risk of stroke, potentially linked to increased cerebral vascular fragility. This should be added and discussed as a potential long-term risk.

Response: We thank the reviewer for raising this point. While some studies have reported associations between very low saturated fat intake and stroke risk, we believe these findings require nuanced interpretation. Several prospective cohort studies have shown that the health effects of reducing saturated fat depend importantly on the replacement nutrient; substituting refined carbohydrates versus unsaturated fats for saturated fats is associated with opposite effects on CVD risk. Moreover, we do not consider the evidence on very low saturated fat diets and stroke risk sufficiently consistent to warrant discussion in our paper. Finally, what constitutes a "very low" saturated fat diet is itself open to debate. The saturated fat content of our WFPB intervention (6.5% of energy) is consistent with several guidelines, which generally recommend limiting saturated fat to ≤10% of energy, with no established minimum, and the American Heart Association recommends ≤6% of energy for LDL-C lowering. In this context, we believe that adding discussion suggesting a potential long-term stroke risk related to the saturated fat level achieved in our intervention would be an overinterpretation of our data.

Lines 530-534: This is the crux of the limitations. Needs more discussion; for example, could similar findings have been seen on meds?

Response: We agree that evaluating diet in the absence of concomitant cholesterol-lowering medication may limit the generalizability of our findings, and potential interaction between diet and medication warrants further discussion. We modified the discussion to now comment on this aspect at two instances.

Please see line 232: *"The decision to test the effect of diet without concomitant use of cholesterol-lowering medication was driven by the objective of rigorously isolating the full potential of diet in improving LDL-C and other CVD risk factors in genetically confirmed HeFH—a question that had not been addressed to date. The absence of cholesterol-lowering medication use among participants may limit the generalizability of our findings, given the central role of these medications in HeFH management. Future studies should determine the incremental LDL-C–lowering effect of diet on top of cholesterol-lowering medication, as well as whether medication type (e.g., statin, ezetimibe, PCSK9 inhibitor), alone or in combination, modifies the effect of diet on LDL-C lowering. Nevertheless, nearly all prior studies using an adequate 2×2 factorial design have reported additive benefits of diet and statins on atherogenic plasma lipids, meaning that concomitant medication use does not attenuate the effects of diet on LDL-C.[33-37]"*

Please see line 251: *"Regarding the cholesterol-lowering mechanisms of diet, measurements of noncholesterol sterols suggest that the WFPB diet, compared to the SAD, reduced plasma LDL-C levels primarily by decreasing cholesterol absorption. As expected, cholesterol synthesis increased to maintain homeostasis; however, the rise in the lathosterol-to-cholestanol ratio indicates that the reduction in*

absorption outweighed the increase in synthesis. These findings aligns with the known effects of increased soluble fiber intake, which forms a gel that traps bile acids, reducing their reabsorption and thereby limiting intestinal cholesterol uptake.[40] This depletion of bile acids promotes hepatic conversion of cholesterol into bile acids, further enhancing reductions in circulating LDL-C.[41] The modulation of cholesterol homeostasis induced by diet is also consistent with that induced by ezetimibe, which lowers LDL-C by inhibiting cholesterol absorption through the Niemann-Pick C1-Like 1 protein.[42] Accordingly, the effect of the WFPB diet on the lathosterol-to-cholestanol ratio further supports the need for comprehensive studies examining how diet interacts with different classes and combinations of cholesterol-lowering medications."

Lines 535-542: Delete. You had absolutely no power, nor any long term follow-up, to understand the potential for underdiagnosis. A difference of 1 or 2 people out of 10 has zero implications for diagnostic thresholds.

Response: We removed the text in the discussion as requested.

Lines 543-546: delete, simply repeating the results; and these findings could very likely be chance.

Response: We removed the text in the discussion as requested.

Line 551-552: delete. absence of significant effect modification (failure to reject null) is not the same as confirmation of beneficial effects (confirming the alterative hypothesis).

Response: We removed the text in the discussion as requested.

Lines 558-658: delete. See earlier comments.

Response: We removed the text in the discussion as requested.

Lines 577-588: delete, highly speculative.

Response: We removed the text in the discussion as requested.

Line 589: delete "important", there is no evidence this was "important." Delete repetition of results text, e.g. lines 590-591, throughout the discussion. Line 591-593: speculative, soften language. Line 593-594: change to "However, the lack of significant effect on GLP-1 does not support this effect on appetite."

Response: Done.

Please see line 264: *"In this study, we observed reduction in perceived appetite sensations with WFPB compared to SAD. The lower energy density of the WFPB may have contributed to this effect, especially considering that the diets were provided in isocaloric, weight-maintaining conditions. However, no evidence of effect on fasting total GLP-1 was found to provide biological support for these results. Future studies should assess how WFPB diets impact total and active GLP-1 in the postprandial state, since fasting measures may miss meal-stimulated differences relevant to perceived appetite sensations."*

Lines 594-599 delete, highly speculative.

Response: Done.

Line 600: change “clinically meaningful” to “possible”. Delete repeated results text, which s most of the rest of the paragraph.

Response: Done. We also modified the paragraph to avoid repeating the results.

Please see line 270: *“Finally, data on participants’ appreciation of the experimental diets provide possible insights into how such a dietary transition could be supported in real-life settings. We hypothesize that the similar appreciation of the WFPB and SAD after each 4-week period may be partly explained by the fact that both diets included typically Canadian meals. This suggests that adapting familiar traditional recipes to fit a WFPB pattern, may help overcome common barriers related to taste acceptability and perceptions of whole food plant-based eating, without reducing overall appreciation.[43-45]”*

Line 625-626: only a limitation, not a strength; edit.

Response: We modified the text accordingly.

Please see line 295: *“Finally, implications associated with the fact that the trial was conducted without cholesterol-lowering medication is a limitation and have been previously discussed.”*

Line 634: suggest delete comment on inter-individual variability (you have not shown this is due to anything other than chance, which is a normal part of the world).

Response: Done.

Line 620: delete.

Response: Done.

Minor comments:

Lines 134-143: “strictly forbidden”, “not allowed” , “only allowed” , “had to”, “not permitted” all need editing. These were free-living participants, they may have been advised or instructed but cannot have been forbidden, not allowed, or forced.

Response: We thank the reviewer for its recommendation. We agree that such vocabulary was not appropriate. We edited the paragraph accordingly.

Please see line 365: *“Participants were instructed to consume only the provided foods and to consume all meals as supplied. They were also asked to avoid consuming any other foods or caloric beverages, including alcohol, during the dietary interventions and to avoid adding any calorie-containing seasoning or salt to their meals. They were instructed to only drink, in addition to the beverages provided, water or caffeine-free diet beverages and a maximum of 2 cups per day of tea, black coffee or other caffeine-containing diet beverages. They were instructed on which calorie-free seasonings to use if desired. A checklist was provided to the participants to identify foods that they had or had not consumed and was used to assess compliance to the dietary interventions. Participants were also instructed to indicate in the checklist if they consumed any non-study foods or beverages. Finally, participants were asked to*

maintain their usual physical activity throughout the entire study and to avoid use of vitamin supplements or natural health products during the trial."

Lines 218-263: these were secondary or exploratory outcomes; move text to supplementary materials.

Response: Done.

Please see line 445: *"Protocols for measurements of appetite hormones, PCSK9, noncholesterol sterols, as well as for the assessment of subjective appetite sensation and appreciation of the diets are presented in the* ***Supplementary Methods****."*

Results, first paragraph: The % who stopped prior meds (and which meds) should be specified in the text.

Response: Done.

Please see line 93: *"Prior to medication discontinuation for the trial, 22% of participants were taking statin monotherapy, 46% were taking statin and ezetimibe, 24% were using a PCSK9 inhibitor with or without concomitant oral therapy, and 8% were not using cholesterol-lowering medication."*

Reviewer 3

The objectives, rationale and study design are sound. The study has been well conducted, and appropriate statistical methods have been applied and competently executed in a transparent manner. The trialists have done well to achieve compliance and encourage patients to complete the study with very few missing observations or patient dropouts.

The paper is worthy of publication with only minor edits which I outline below.

Response: We would like to thank the Reviewer for the positive appreciation of our work.

In the title and elsewhere please state the number of periods and treatments used in the crossover design, either in full as "two-period, two-treatment crossover", or as an "AB/BA crossover" design.

Response: Done. We added the information where relevant in the paper.

Please see the title: *"Impact of a whole food, plant-based diet on LDL-cholesterol and cardiovascular risk factors in adults with heterozygous familial hypercholesterolemia: a randomized, two-period, two-treatment, crossover, fully controlled feeding trial".*

On line 24 of the abstract under methods please quote the number of patients assigned to each treatment sequence, e.g., " .. confirmed HeFH, free of cholesterol-lowering medication, consumed a WFPB and a SAD for 4 weeks each in a random order, (27 to receive SAD first and 23 WFPB first), under fully controlled, isocaloric feeding conditions."

Response: Done.

Please see line 22: *"In this randomized, two-period, two-treatment, crossover, controlled feeding trial, 50 adults with genetically confirmed HeFH, free of cholesterol-lowering medication, consumed a WFPB and a SAD for 4 weeks each in a random order (27 to receive SAD first and 23 WFPB first), under fully controlled, isocaloric feeding conditions."*

The abstract should also identify the primary as opposed to secondary outcome/objective. This can be done succinctly, by modifying line 21 under the heading Objective to "(SAD), on the primary outcome of LDL-cholesterol (LDL-C), and other CVD risk factors".

Response: Done.

Please see line 20: *"This study aims to evaluate the impact of a WFPB, in place of a standard American diet (SAD), on LDL-cholesterol (LDL-C) (primary outcome) and other CVD risk factors among adults with HeFH."*

The abstract should state or reference the source of funding.

Response: Done.

Please see line 36: *"(clinicaltrials.gov registration: NCT05181553; funding: Canadian Institutes of Health Research)."*

In the main text under the section "Study design" add a sentence to explain the rationale for using a cross-over design, e.g., something along the lines of the following, perhaps with a scientific reference. "A cross-over design is more efficient way of measuring a treatment effect as each patient acts as their own control and a smaller sample size is required. The study laboratory parameters are expected to be affected relatively quickly by changes in diet, and a 2-to-4-week wash-out period is expected to more than adequate to avoid a carry-over effect."

Response: We added the suggested text within the first two paragraphs of the "Study design" section.

Please see line 334: "*A crossover design is an efficient approach for estimating treatment effects because each participant serves as their own control, thereby reducing between-participant variability and the required sample size.*[48]*"*

Please see line 353: *"Four-week periods of fully controlled feeding are well established to produce significant changes in plasma LDL-C and other outcomes of interest, as shown in numerous previous studies.*[15,24,53] *Likewise, a two- to four-week washout period has also been shown to be sufficient to minimize potential carryover effects between periods.* [15,24,53]*"*

The choice of a linear mixed model with a random intercept for each patient is a good one, as it allows for correlations between observations on the same patient with each acting as their own control; the choice of the restricted maximum likelihood to fit these models is also optimal. In fact, the methods section should include a more precise description of the models, e.g., "Linear mixed models were fitted to the primary and secondary outcomes using restricted maximum likelihood with a random intercept term for each patient to allow for correlation between observations on the same patient so that each patient acts is its own control."

Response: We thank the reviewer for his remark. We edited the second paragraph of the statistical analysis section accordingly.

Please see line 474: *"We first validated protocol adequacy by comparing compliance to each diet, mean daily energy intake during each diet, post-intervention weight and visceral adipose tissue mass, as well as weight change during each diet, using mixed models for repeated measures with the packages lme4 (v1.1.35.5) and lmerTest (v3.1.3) with restricted maximum likelihood. Diet was treated as a fixed effect, while subjects were treated as a random effect (random intercept) to account for within-participant correlation, so that each participant served as their own control. The models were adjusted for BMI (kg/m^2) and sequence (SAD-WFPB, WFPB-SAD)."*

It is worth stating that the linear mixed models allow for unbalanced data and for patients missing a data point to be retained in the analysis.

Response: We thank the reviewer for this suggestion. However, because there were no missing data and the data were balanced given the crossover design, we believe that adding these details would be unnecessary and potentially confusing.

Although page 15 mentions a period*diet interaction, please make it clear in the methods that an apriori decision was made to adjust for the period effect so that this is clear at a glance.

Response: We now added a mentioned that this decision was made a priori.

Please see line 543: *"For these analyses, assessment period (first week vs last week) was treated as a fixed effect, and participants were treated as a random effect. To determine the difference between the dietary interventions, an interaction term (assessment period * diet) was added to the models a priori."*

Please include a table, at least in the supplement, that summarises the data with two rows for the two treatment sequences AB , BA and columns from left to right for the number of patients at the start , the number of observations (patients with measurements), Mean (SD) of the LDL-c readings at the start and end of each period and the change in LDL-c across the period.

Response: We thank the reviewer for this suggestion. We did not add the requested table because we believe it could create confusion regarding the effect of interest in our study. The contrast of interest was LDL-C after the SAD versus LDL-C after the WFPB (same contrast for all other outcomes). LDL-C values measured at the beginning of each diet period are not informative for the primary contrast of interest, because participants were not under controlled dietary conditions when these measurements were obtained. Importantly, our analysis does not compare within-diet changes in LDL-C, but rather post-diet differences between the two interventions. We consider that the main tables, together with the table reporting LDL-C levels by sequence, already provide sufficient transparency and are fully aligned with the crossover design of our study.

Two minor points.
Please provide a more detailed sentence on how the stratified randomisation was carried out.

Response: We added details on the randomization.

Please see line 346: *"Randomization was performed by the research coordinators and was stratified by sex (female, male) and LDLR genotype (RN vs. others), using eight blocks of eight participants and a 1:1*

allocation ratio, such that each block included equal numbers of females and males and equal numbers of individuals with the RN genotype and other genotypes."

The study sample size is adequate and appears correct, but for completeness please specify the precise input/options used in the G*power sample size calculation.

Response: We added a table in the supplementary material presenting all input parameters for our power calculation.

Please see **Supplementary Table 10**.

We would like to thank the editorial team of Nature Communications and the reviewers for their constructive comments. We have been able to address all the reviewers' concerns and make the appropriate changes in the revised manuscript. Please find below our point-by-point response to comments. All authors agree with the changes made to the paper.

Reviewer #2 (Remarks to the Author):

For authors:

The authors partly responded to some of the concerns, but unfortunately not to other major comments. For example, they continue to equivocally present null findings and ignore potential risks in their discussion. Attention to these issues is needed.

Major comments:

Introduction: As noted previously, some references are described incorrectly. Ref 14 does not show that a diets low in red and processed meats and high in minimally processed plant foods positively affects CVD risk factors. In fact, it shows minimal and inconsistent effects of red meat consumption on CVD risk factors. The latter finding needs to be specified (not ignored or deleted) and presented accurately.

Response: We thank the reviewer for this comment. In the original submission, reference 14 corresponded to the paper by Guasch-Ferré et al., published in Circulation in 2019: Meta-Analysis of Randomized Controlled Trials of Red Meat Consumption in Comparison With Various Comparison Diets on Cardiovascular Risk Factors (https://www.ahajournals.org/doi/10.1161/CIRCULATIONAHA.118.035225). In the revised manuscript submitted after the first round of review, this paper became reference 13, whereas reference 14 became the paper by Yokoyama et al. on plant-based diets and plasma lipids. Our understanding is that the reviewer's comment still refers to the Guasch-Ferré et al. paper.

In the first round of review, the reviewer noted: "Lines 57–59: These references are likewise presented incorrectly. Ref 14 does not show that a diet low in red and processed meats and high in minimally processed plant foods positively affects CVD risk factors. In fact, it shows minimal and inconsistent effects of red meat consumption on CVD risk factors. This citation is important and needs to be presented accurately."

The original sentence was: "In the shorter term, randomized clinical trials (RCTs) have demonstrated that these diets positively affect CVD risk factors, including atherogenic plasma lipids.[14,15]"

In response to this comment, we acknowledged that the original formulation was too broad and revised the sentence as follows: "In the shorter term, randomized controlled trials (RCTs) have shown that such dietary patterns, or some of their components, such as replacing red meat with high-quality plant protein sources (e.g., legumes, soy, and nuts), positively affect CVD risk factors, including atherogenic plasma lipids.[13–16]"

In this revised wording, reference 13 (Guasch-Ferré et al.) supports specifically the statement regarding replacement of red meat with high-quality plant protein sources. As stated in Guasch-Ferré's abstract, "*relative to high-quality plant protein sources, red meat yielded lesser decreases in total cholesterol and LDL cholesterol*", and the authors concluded that "*substituting red meat with high-quality plant protein sources, but not with fish or low-quality carbohydrates, leads to more favorable changes in blood lipids and lipoproteins*." In the methods and clinical perspective section, high-quality plant protein sources are defined as legumes, soy, nuts, and other plant protein sources.

We therefore believe that our revised interpretation appropriately reflects the findings of Guasch-Ferré et al. Specifically, the paper supports the point that the lipid effects of red meat depend on the comparison diet, and that substitution with high-quality plant protein sources is associated with more favorable lipid

changes. This is the aspect of the study that is relevant to our manuscript, given that our trial compares a whole-food, plant-based diet aligned with Canada's Food Guide, low in red meat and rich in minimally processed plant foods including legumes, soy, and nuts, with a standard American diet designed to reflect average intakes in Canadian and US adults.

To further address the reviewer's concern and avoid any possible misinterpretation, we revised the text to align even more closely with the wording and conclusions of the Guasch-Ferré et al. paper. In the revised version, this is now reference #16. The sentence now reads (line 57):

"In the shorter term, randomized controlled trials (RCTs) have shown that such dietary patterns positively affect CVD risk factors, including atherogenic plasma lipids.[13-15] Notably, a meta-analysis of RCT of red meat consumption in comparison with various comparison diets reported that substituting red meat with high-quality plant protein sources (e.g., legumes, soy, and nuts) leads to more favorable changes in blood lipids and lipoproteins.[16]"

Methods:

As noted previously, the exclusion of adults age 60+ needs justification. The authors provided an explanation in the response letter but not in the actual manuscript. The justification should be added: "The age threshold was driven by the requirement to discontinue cholesterol-lowering medication. Given the higher baseline CVD risk in older adults, we excluded participants aged ≥60 years as a risk-mitigation measure, based on safety considerations related to temporary treatment withdrawal."

Response: We revised the text.

Please see line 331: *"The age threshold was driven by the requirement to discontinue cholesterol-lowering medication. Because older adults have a higher baseline CVD risk, participants aged ≥60 years were excluded as a risk-mitigation measure based on safety considerations related to temporary treatment withdrawal. Other exclusion criteria included conditions that exacerbate CVD risk (e.g., homozygous FH, personal history of CVD, diabetes or use of anti-diabetic medication, severe obesity, unstable body weight over the previous 3 months, uncontrolled hypertension, and genetic hypertriglyceridemia), allergies or aversions to components of the experimental diets, and any condition that could interfere with optimal participation in the intervention."*

As noted previously, many different outcomes and analyses are listed (lines 279-368). The text should clearly specify, in once place, which of these were prespecified primary, secondary, or exploratory outcomes; and which were post-hoc. (some are noted as a priori; many others are not specified). The authors point to supplementary text, but it does not contain this information. The text should clearly specify, in the main paper, which of the outcomes were prespecified primary, secondary, or exploratory outcomes; and which were post-hoc

Response: We added the information in a single place, at the beginning of the statistical analyses section. We reformulated some sentence throughout this section to avoid repetition.

Please see line 472: *"LDL-C was the primary outcome of the trial. All other outcomes were considered exploratory. Among these exploratory outcomes, the following were prespecified: protocol adequacy; other CVD risk factors; 10-year CVD risk; modification of the effect of diet on LDL-C according to sex, LDLR genotype, and diet sequence; and sex-specific perceived appetite sensations and meal appreciation, as well as changes in the latter two outcomes. Post hoc exploratory outcomes included effect modification by age; the proportion of participants whose post-diet LDL-C levels exceeded thresholds for FH investigation; plasma PCSK9; appetite hormones; noncholesterol sterols; and correlates of dietary response."*

Results:

As noted previously, the authors should be clear about results that were not statistically significant. Lines 141-143 should read, "The proportion of participants whose LDL-C levels exceeded the thresholds warranting investigation… was not significantly different on the WFPB diet versus the SAD diet (79.2% versus 87.5, P=0.13). As before, I strongly recommend deleting the associated supplementary figure, which is not needed and unnecessarily emphasizes a nonsignificant findings

Response: We modified the text to be clear about statistical significance.

Please see line 144*: "There was no evidence of a statistically significant difference at the end of the WFPB and the SAD in the proportion of participants whose LDL-C levels remained above the thresholds warranting FH investigation according to the Simplified Canadian Definition for FH (≥4.50 mmol/L for those aged 18 to 40 years and >5.00 mmol/L for those aged over 40 years), with corresponding proportions of 79.2% and 87.5% (P=0.13)."*

We also removed the figure from the supplementary material.

Similarly, for the effects on GLP-1 or PYY, the text should specify that no significant effects were seen (rather than just reporting the differences)

Response: We modified the text to be clear about statistical significance.

Please see line 157: *"Regarding appetite hormones, no evidence of statistically significant effects of the WFPB relative to the SAD was observed on fasting total GLP-1 (3.4%, 95% CI = –0.6% to 7.4%; P = 0.09) and peptide YY (–2.6%, 95% CI = –7.0% to 1.7%; P = 0.23)."*

Line 160: As noted previously, a single correlation with P=0.04 among many exploratory findings is unlikely to be meaningful and more likely a chance finding. Using declarative text "was associated with" is not appropriate here, without further caveats. Add here: "… P = 0.04), although this exploratory finding was not adjusted for multiple comparisons and may have been due to chance."

Response: We added a clear statement indicating that the *P* value was not adjusted for multiple comparisons.

Please see line 163: *"For ApoB response, each 1 pmol/L-increment in fasting total GLP-1 was associated with a –0.15% decrease in ApoB diet-induced changes (95% CI = –0.31% to –0.01%, P value not adjusted for multiple comparisons = 0.04). The partial R^2 associated with fasting total GLP-1 was 12.6%."*

We agree that this exploratory finding should be interpreted with caution. However, because the suggested wording *"although this exploratory finding was not adjusted for multiple comparisons and may have been due to chance"* is interpretative rather than descriptive, we did not add it to the Results section. To address concerns of the reviewer with regard to our choice to not adjust for multiple comparisons, we added the following in the strengths/limitations section of the discussion:

Please see line 300: *"Sixth, given the number of exploratory outcomes assessed, some findings may have occurred by chance."*

As noted previously, very low SFA diets have been consistently associated with risk of stroke, potentially linked to increased cerebral vascular fragility. This has been seen irrespective of the replacement nutrient. The diet used here is in the range where higher risk was identified. This literature and potential risk cannot be ignored. This should be added to the discussion as a potential long-term risk.

Response: We thank the reviewer for the suggestion. After reviewing the evidence in detail, we do not think it supports the statement that very low SFA diets have been consistently associated with higher stroke risk. We also do not consider the dietary intervention tested in our study to represent a very low-

SFA diet. Accordingly, we do not think this concern should be discussed in our manuscript as an established long-term risk of the intervention tested, for the reasons outlined below.

In our view, if this association were consistent, this would be expected to emerge across meta-analyses of prospective cohort studies, with higher SFA intake being associated with lower stroke risk such that very low SFA intake would represent the higher-risk reference category. With this in mind, we reviewed the key meta-analyses published over the past approximately 15 years, beginning with the seminal work by Siri-Tarino et al. published in the American Journal of Clinical Nutrition in 2010 (https://www.sciencedirect.com/science/article/pii/S0002916523016726?via%3Dihub). This meta-analysis reported no evidence of an association between SFA intake and stroke risk (RR: 0.81; 95% CI: 0.62, 1.05; P = 0.11). Of the 8 studies included for stroke, 2 examined hemorrhagic stroke exclusively. When these 2 studies were excluded, the pooled RR (95% CI) was 0.86 (0.67, 1.11). Likewise, exclusion of 2 studies conducted in Japan, where habitual SFA intake is known to be substantially lower than in Western populations, did not materially alter the estimate (RR: 0.90; 95% CI: 0.70, 1.15).

Following Siri-Tarino et al., De Souza et al. published a meta-analysis in BMJ in 2015 (https://www.bmj.com/content/351/bmj.h3978). Based on 12 studies, they reported no association between SFA intake and ischemic stroke, with a summary multivariable-adjusted risk ratio of 1.02 (95% CI: 0.90, 1.15; P = 0.79). Excluding four comparisons whose risk estimates were derived from the earlier Siri-Tarino meta-analysis did not materially change the findings (RR: 1.03; 95% CI: 0.89, 1.19; P = 0.68).

In 2018, Muto and Ezaki published another meta-analysis of prospective cohort studies, this time focusing specifically on stroke subtypes (https://pdfs.semanticscholar.org/400b/ada37e882b9ce531ed4c92d72f360b55f901.pdf). For intracerebral hemorrhage excluding subarachnoid hemorrhage, they observed a strong inverse association in Japanese studies (n = 3; HR: 0.55; 95% CI: 0.32, 0.94), but not in non-Japanese studies (n = 2; HR: 0.98; 95% CI: 0.62, 1.53). For ischemic stroke, they reported a modest inverse association in Japanese studies (n = 4; HR: 0.82; 95% CI: 0.71, 0.93), but again not in non-Japanese studies (n = 7; HR: 0.93; 95% CI: 0.84, 1.03). Their conclusion was: *"In Japanese but not in non-Japanese, a diet high in saturated fat is associated with a low risk of intracerebral hemorrhage and ischemic stroke. This may be due to differences in the range of intake of saturated fat, genetic susceptibility, incidence of lacunar infarction, and/or confounding factors such as dietary proteins."*

To the best of our knowledge, the most recent meta-analysis of prospective cohort studies was published by Kang et al. in Nutrition, Metabolism & Cardiovascular Diseases in 2020 (https://www.nmcd-journal.com/article/S0939-4753%2819%2930380-1/abstract). Unlike the previous meta-analyses, this study reported that higher SFA intake was associated with lower stroke risk, which is more aligned with the reviewer's interpretation. This meta-analysis included 2 additional studies relative to prior work: (1) the PURE cohort, which reported a strong inverse association between SFA intake and stroke risk in approximately 135,000 adults from multiple developing countries, and (2) the EPIC-NL cohort, which reported no association between SFA intake and stroke risk. Accordingly, the difference in conclusions between Kang et al. and earlier meta-analyses appears to be driven largely by the inclusion of the PURE study.

Importantly, subgroup analyses in Kang et al. also showed marked regional differences. Higher SFA intake was associated with lower stroke risk in Asian studies, but not in European or North American studies (PURE was not included because of its multinational design). Notably, the 5 Asian studies were all conducted in Japan and were the same studies emphasized in the previous meta-analysis by Muto and Ezaki. In our view, this further supports the interpretation that the inverse association has been observed primarily in Japanese cohorts rather than consistently across populations.

Meta-analyses of randomized controlled trials evaluating SFA reduction in relation to stroke are also relevant. In the 2020 update of their Cochrane review (https://www.cochranelibrary.com/cdsr/doi/10.1002/14651858.CD011737.pub2/full), Hooper et al. concluded that the effects of reducing saturated fat intake on stroke were unclear because the certainty of evidence was very low. More recently, Steen et al. published a similar analysis in Annals of Internal Medicine on December 16, 2025 (https://www.acpjournals.org/doi/10.7326/ANNALS-25-02229). In that study, the RR for fatal and nonfatal stroke associated with reducing SFA intake was 0.83 (95% CI: 0.58, 1.19). When stratified by baseline cardiovascular risk, reducing SFA intake appeared to have little or no effect on stroke in individuals at low cardiovascular risk, whereas it may confer benefit in those at high cardiovascular risk.

Taken together, and considering both prospective cohort studies and randomized trials, we do not believe the available evidence supports the statement that "very low SFA diets have been consistently associated with higher risk of stroke." Rather, the most reproducible signal appears to be confined largely to Japanese cohorts.

We also do not consider dietary habits in Japan to be comparable to those tested in our trial, namely a whole-food, plant-based diet aligned with Canada's Food Guide, compared with a standard American diet designed to reflect average intakes in Canadian and US adults. In our whole-food, plant-based diet, SFA contributed 6.5% of total energy intake. With a mean energy intake of 2770 kcal/day (Supplementary Table S1), this corresponds to approximately 20 g/day of SFA. In the Japanese studies that contributed substantially to the signal discussed above, SFA intakes in the lowest and highest quantiles were 7.2 and 15.4 g/day (Seino, 1997), 5.2 and 17.1 g/day (Iso, 2003), 7.0 and 21.0 g/day (Sauvaget, 2004), 9.2 and 19.8 g/day (Yamagishi, 2010), and 9.6 and 24.9 g/day (Yamagishi, 2013). In PURE (Deghan, 2017), median SFA intake was 8% with Q1 = 2.8% and Q5 = 13.2%; all these values were extracted from Table 1 of Kang et al. (2020). Thus, the SFA content of our whole-food, plant-based diet was similar to the upper range of intake observed in these Japanese cohorts and was closer to the median than Q1 in PURE. On that basis, we do not consider the SFA content of our intervention diet to be "very low," nor do we consider it directly comparable to the lower intake ranges that may have contributed to the inverse associations observed in those studies.

Finally, we also reviewed recent guidance documents from the Canadian Cardiovascular Society (2021), the European Society for Cardiology (2021; https://onlinecjc.ca/article/S0828-282X(21)00165-3/fulltext), the National Lipid Association (2023; https://www.lipidjournal.com/article/S1933-2874(23)00185-X/fulltext) and the 2026 American Heart Association scientific statement, Dietary Guidance to Improve Cardiovascular Health, published on March 31, 2026 (https://www.ahajournals.org/doi/10.1161/CIR.0000000000001435). To our knowledge, all advocate for replacing SFA in the diet by PUFAs, MUFAs, and/or carbohydrates from whole grains, and none cautioned against a possible increase in stroke risk or cerebral vascular fragility associated with very low SFA intake. If the evidence for such a concern were considered sufficiently robust, consistent, and generalizable, we would expect it to be acknowledged in at least some of these documents.

For these reasons, we do not think it would be appropriate to revise the Discussion in a way that presents increased stroke risk as an established or likely long-term consequence of the dietary intervention tested in our study.

Abstract: My comment to add the body weight change is not based on either its presence or lack of clinical or statistical significance. This is a relevant outcome to interpret all the findings, and should be included in the abstract.

Response: We added the following on line 27: *"Body weight decreased by 0.82% (95% CI: 0.37%, 1.27%) during the SAD and by 1.30% (95% CI: 0.88%, 1.73%) during the WFPB. The reduction was*

greater during the WFPB than the SAD (difference: 0.48%; 95% CI: 0.05%, 0.92%; P=0.03), although body weight at the end of each diet was similar (76.4 ± 2.0 vs 76.3 ± 2.0 kg; P=0.45).”

We had to made small edits in the abstract to keep the word count at its minimal. This explains the other changes made to the abstract.
